# Soybean Auxin Transporter PIN3 Regulates Nitrate Acquisition to Improve Nitrogen Use and Seed Traits

**DOI:** 10.1002/advs.202511907

**Published:** 2025-11-18

**Authors:** Huifang Xu, Shiyu Huang, Jie Wang, Tian Wang, Qingqing Han, Kexin Wu, Zhen Gao, Xiaolei Shi, Tianli Tu, Ming Wang, Laimei Huang, Jiaomei Chen, Yunqi Liu, Yumei Zhang, Guoqiang Lin, Zhichang Chen, Xu Chen

**Affiliations:** ^1^ State Key Laboratory of Agricultural and Forestry Biosecurity College of Life Science Haixia Institute of Science and Technology Fujian Agriculture and Forestry University Fuzhou 350002 China; ^2^ State Key Laboratory for Crop Stress Resistance and High‐Efficiency Production College of Life Sciences Northwest A&F University Yangling 712100 China; ^3^ Hebei Laboratory of Crop Genetics and Breeding Institute of Cereal and Oil Crops Hebei Academy of Agricultural and Forestry Sciences Shijiazhuang 050035 China; ^4^ Zhongguancun Xuyue Non‐invasive Micro‐test Technology Industrial Alliance Beijing 10080 China; ^5^ Institute of Crops Fujian Academy of Agricultural Sciences Fuzhou 350013 China; ^6^ State Key Laboratory of Soil and Sustainable Agriculture Institute of Soil Science Chinese Academy of Sciences Nanjing 211135 China; ^7^ Collaborative Innovation Center of Soybean Biotechnology and Nutrition Efficiency Henan 475001 China

**Keywords:** auxin, leaf development, nitrate, PIN3, seed oil, soybean

## Abstract

Enhancing nitrogen‐use efficiency is essential for boosting crop yields and advancing sustainable agriculture, particularly in the absence of synthetic fertilizers. Despite the inherent nitrogen‐fixation capacity of the staple legume crop soybean (*Glycine max*) by symbiotic rhizobia, improving nitrogen use has been challenging. Here, a role for the auxin‐efflux transporters PIN3a and PIN3b in soybean nitrate acquisition is uncovered. PIN3a/b localizes to the plasma membrane, and high environmental nitrate induces PIN3a degradation and its accumulation at cell junctions. Disrupting *PIN3* homologs results in auxin over‐accumulation, impairs pavement‐cell polarity, and enhances signaling via the transcription factors *ARF* and *STF3/4*. These transcription factors separately bind to and activate the *NPF2.13* promoter, thereby strengthening nitrate uptake. *pin3ab* and *pin3abd* mutants have enhanced nitrate acquisition and resistant to high nitrate on pavement‐cell growth. The elevated nitrogen accumulation translates to higher oil contents in *pin3ab* mutant seeds in an elite cultivar background across multiple years and field locations. The findings shed light on the regulation of nitrate uptake in crop‐plant development and demonstrate the unexpected potential of manipulating auxin transporters to enhance soybean nitrogen‐use efficiency and agronomic performance.

## Graphical abstract

Xu et al. discovers that switching off auxin transporters in soybean, PIN3a and PIN3b, disrupts auxin flow; this triggers ARF‐STF3/4 signal cascade to activate the nitrate importer *NPF2.13* and soil nitrogen acquisition. Multi‐year field trials show edited soybeans maintain yield with higher oil content, offering a potential genetic route to improve nitrogen efficiency and seed quality.

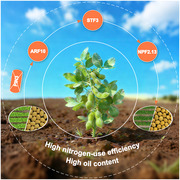

## 1 Introduction

As a constituent of chlorophyll, polyamines, nucleic and amino acids, nitrogen is an essential macronutrient for plant growth and development. Provision of synthetic nitrogenous fertilizers to crops is a widespread yet fraught strategy to boost production, despite the average nitrogen fertilization of all crops increasing from ≈44.6 kg ha^−1^ in the 1960s to ≈100.9 kg ha^−1^ in the 2010s.^[^ ^1^ ^]^ However, only ≈50% of applied nitrogenous fertilizers are effectively absorbed and used by crops.^[^ ^2^ ^]^ This inefficiency not only caps crop production, but also has detrimental effects on the environment, including ammonia volatilization, denitrification, and nitrate leaching. These processes account for losses of 10–60% of nitrogen fertilizer.^[^ ^3^ ^]^ Given these challenges, there is an urgent need to develop crops with enhanced nitrogen‐use efficiency.

Nitrogen‐use efficiency is collectively determined by nitrogen uptake, transport, assimilation, and remobilization.^[^ ^4^ ^]^ External nitrate levels are monitored, and internal demand is coordinated to adjust nitrate metabolism via transporters.^[^ ^5^ ^]^ NITRATE TRANSPORTER 1 (NRT1)/PEPTIDE TRANSPORTER (PTR) families (NPFs) move nitrate from roots to other tissues using high‐ and low‐affinity systems depending on soil nitrate concentrations.^[^ ^6^ ^]^ Plant genomes encode numerous *NPFs*, with Arabidopsis NPF6.3 the best‐studied dual‐affinity nitrate transporter^[^ ^7^ ^]^ that also participates in cell‐to‐cell auxin transport.^[^ ^8^ ^]^ Under low‐nitrate conditions, auxin and NPFs interact to modulate root architecture by promoting basipetal auxin transport and inhibiting its accumulation in the lateral root tip, thereby suppressing lateral‐root growth.^[^ ^8^ ^]^ Nitrate triggers phosphorylation of the auxin transporter PIN‐FORMED 2 (PIN2), with dephosphorylated PIN2^Ser439^ unable to respond to external nitrate.^[^ ^9^ ^]^ NRT2.1 physically interacts with PIN7, resulting in the suppression of auxin efflux specifically under low‐nitrate conditions for control of root growth.^[^ ^10^ ^]^ NIN‐LIKE PROTEIN 7 (NLP7), a master transcriptional regulator of the high‐nitrate response, enhances cytokinin biosynthesis and auxin transport in roots, through direct upregulation of *PIN* transcripts.^[^ ^11^ ^]^

In leaves, ≈80% of nitrogen is sequestered within chloroplasts.^[^ ^12^ ^]^ This leads to a positive correlation between chlorophyll content and nitrogen concentration. Nitrogen starvation reduces leaf size, attributable to diminished light‐use efficiency and carbon assimilation.^[^ ^13^ ^]^ Beyond nitrogen's role in photosynthesis, leaf growth is a precisely orchestrated cellular process, balanced between cell expansion and proliferation.^[^ ^14^ ^]^ While nitrogen is recognized for its role in stimulating leaf growth, the molecular pathways through which it operates have been largely unclear. Nitrogen signaling accelerates cell‐cycle progression and endoreplication,^[^ ^15^ ^]^ and cell elongation with the input of cytokinins.^[^ ^16^ ^]^ These findings underscore the multifaceted influence of nitrogen on plant growth and development, implying the complexity of the signaling pathways involved.

Nitrate (NO_3_^−^) and ammonium (NH_4_^+^) are the primary sources of inorganic nitrogen, with Arabidopsis and soybean preferring NO_3_^−^ while rice favors NH_4_^+^.^[^ ^17^, ^18^ ^]^ Soybean obtains an impressive 60–70% of its nitrogen needs from symbiotic nitrogen fixation, a process that converts atmospheric dinitrogen into ammonia compounds, thereby providing a usable nitrogen source.^[^ ^19^ ^]^ Substantial effort has focused on improving nitrogen fixation in soybean and other legumes, yet nitrogen‐use efficiency itself has been relatively overlooked.

Here, we investigated the impact of auxin transport on nitrogen acquisition in soybean and found that disruption of auxin transport unexpectedly enhanced nitrate uptake. High environmental nitrate promotes leaf‐cell expansion by increasing intracellular auxin levels as a consequence of PIN3a and PIN3b degradation. Auxin over‐accumulation stimulates nitrate uptake, which is activated via the auxin‐signaling transcription factor AUXIN RESPONSE FACTOR (ARF) and light‐signaling transcription factor TGACG‐MOTIF BINDING FACTOR 3 (STF3, a HY5 homolog). These findings highlight the intersection of PIN3‐mediated auxin transport with nitrate acquisition and light cues for proper soybean growth and enhanced agronomic traits.

## 2 Results

### 2.1 Proper Auxin Transport is Required for Nitrate Uptake

**Figure 1:**
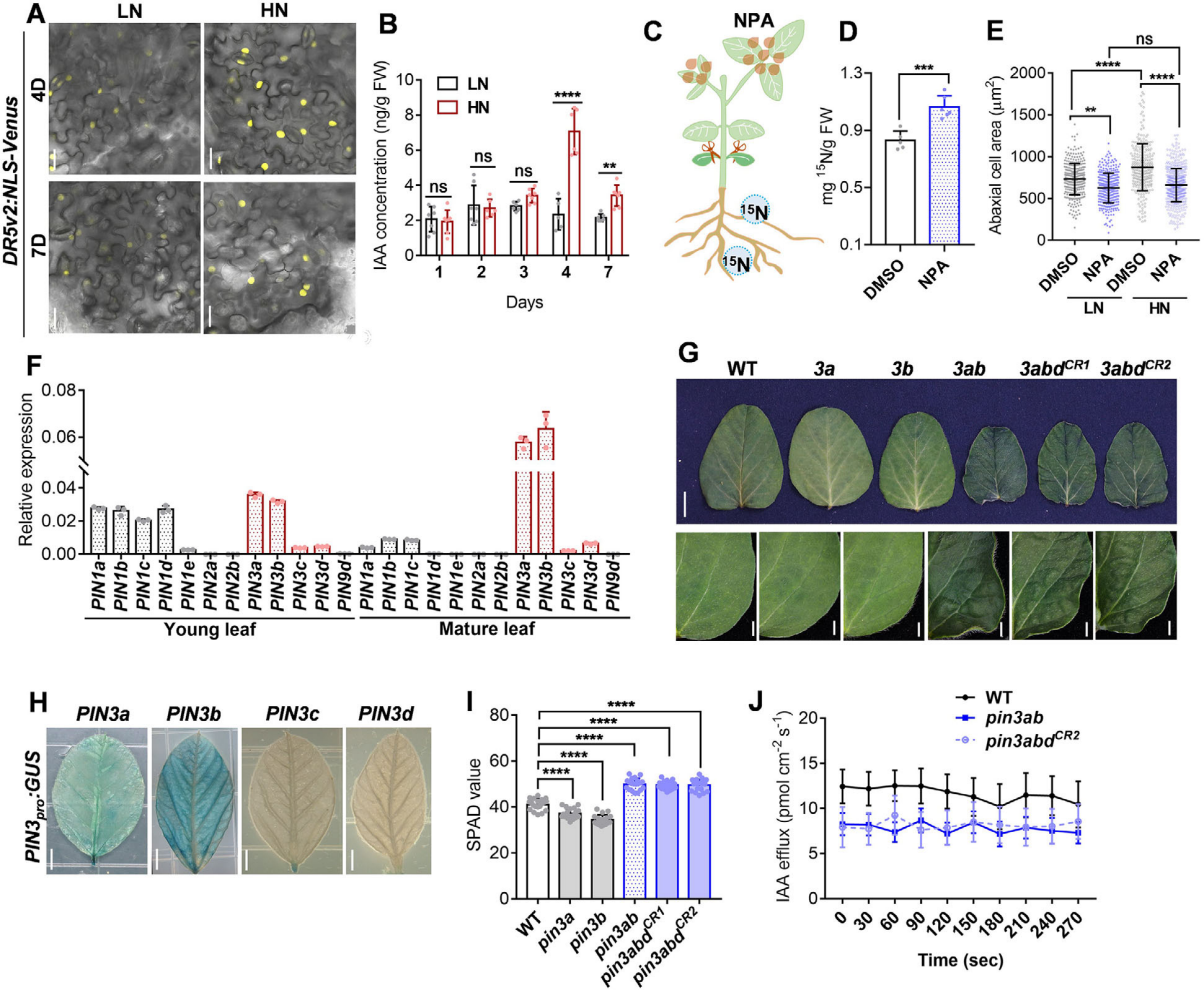
Auxin transport is involved in nitrate‐mediated leaf‐cell expansion. A) Confocal analysis of Venus signal in *DR5v2_pro_:NLS–Venus* plants transferred at 6 days to high‐nitrate (HN) or low‐nitrate (LN) conditions for a further 4 (top) or 7 days (bottom). Scale bar = 20 µm. B) IAA quantification in leaf samples by UPLC–ESI–MS/MS. 6‐day‐old WT plants were grown in high‐ or low‐nitrate conditions and harvested at the indicated number of days thereafter. IAA concentrations were compared between high‐nitrogen and low‐nitrogen conditions on respective days. Data are the mean ± SD of n ≥ 5 replicates. *p*‐values were determined by two‐way ANOVA Turkey's multiple comparisons test (^**^ *p* < 0.01; ^****^ *p* < 0.0001; ns, non‐significant). C,D) Summary of treatment scheme (C) and ^15^N quantification at leaf level (D). WT plants were germinated in vermiculite for 13 days, and the cotyledons were removed;  10 µm NPA was then sprayed on the leaves for 1 day (DMSO‐treated leaves were used as a control). DMSO‐ and NPA‐treated plants were incubated in a 5.3 mm ^15^NO_3_^−^‐containing solution for another 7 h, then all leaves were harvested for ^15^N detection. Data are the mean ± SD of *n* = 5 for DMSO and 6 for NPA. *p*‐values were determined by a two‐tailed Student's *t*‐test (^***^ *p* < 0.001). E) 6‐day‐old WT plants were grown in high‐ or low‐nitrogen conditions for 3 days and then sampled 2 days after treatment with 10 µM NPA. Data are the mean ± SD of *n* = 315, 347, 263, and 484 cell samples for each condition, respectively. *p*‐values were determined by two‐way ANOVA with Tukey's multiple‐comparisons (^**^ *p* < 0.01; ^****^ *p* < 0.0001; ns, non‐significant). F) RT–qPCR of *PIN* expression levels in young (6‐day‐old) or mature (11‐day‐old) leaves. Data are the mean ± SD of *n* = 3, and *ACT11* was used to normalize relative expression. H) Histological analysis of GUS expression from *PIN3a–d_pro_:GUS* reporter constructs in 11‐day‐old leaves. Scale bars = 5 mm. G,I) Leaf phenotypes (G) and SPAD values (I) of 7‐day‐old WT and *pin3a*, *pin3b*, *pin3ab* , and *pin3abd^CR^* mutants cultivated in growth chambers. Scale bars = 1 cm (upper panel) and 20 mm (lower panels) in G. Data are the mean ± SD of *n* = 18–20 leaf samples for each genotype. *p*‐values were determined by one‐way ANOVA Dunnett's multiple comparisons test (^****^ *p* < 0.0001). J) NMT analysis of IAA efflux capacity in 7‐d‐old WT, *pin3ab*, and *pin3abd^CR2^* epidermal leaf cells. Data are the mean ± SD of *n* = 8 cells.

Prevailing theories posit that nitrate increases leaf size by modulating the cell cycle rather than cell expansion.^[^ ^15^ ^]^ Local auxin signaling coordinates apoplastic acidification, thereby activating cell‐wall‐loosening enzymes to enable cell expansion and changes in turgor pressure.^[^ ^20^, ^21^, ^22^ ^]^ To investigate whether auxin contributes to nitrate‐mediated leaf growth in soybean, we treated soybean roots with varying concentrations of nitrate and quantified auxin levels in leaves using ultra‐performance liquid chromatography–mass spectrometry (UPLC–MS). Treatment with 5.3 mm nitrate resulted in the highest auxin accumulation in leaves (Figure S1A, Supporting Information). Consistent with this, we monitored auxin accumulation in mature leaves of stable‐transgenic plants expressing the genetically encoded *DR5v2_pro_:NLS–Venus* reporter. Under high‐nitrate conditions (5.3 mm), Venus fluorescence was stronger after 4 and 7 days of growth compared to that under low‐nitrate conditions (no nitrate added) (**Figure** **1**A). This observation was further corroborated by UPLC–MS analysis, which revealed that plants grown under high‐nitrate conditions had significantly higher indole‐3‐acetic acid (IAA) content after 4 and 7 days of nitrate treatment compared to those grown under low‐nitrate conditions (Figure 1B). These results confirm that nitrate promotes auxin accumulation in soybean leaves.

Auxin levels are intricately coordinated by metabolism, transport, and signaling pathways.^[^ ^23^, ^24^, ^25^ ^]^ Among them, auxin signaling enhances nitrogen‐use efficiency in rice grain yield by promoting nitrogen uptake and assimilation.^[^ ^26^ ^]^ To explore whether auxin transport is implicated in soybean nitrate acquisition, we conducted an experiment on hydroponically grown plants to examine nitrate‐uptake capacity. We applied ^15^NO_3_^−^ to the roots for 7 h and subsequently measured the ^15^N levels in the leaves. In parallel, we treated hydroponically grown plants with high nitrate in the roots to simulate normal fertilizer regimes, while simultaneously applying the auxin‐transporter inhibitor N‐1‐naphthylphthalamic acid (NPA) to leaves. Strikingly, compared to the high‐nitrate treatment alone, the addition of NPA significantly elevated ^15^N contents in leaves (Figure 1C,D). This finding suggests that inhibiting auxin transport in leaves can enhance nitrate uptake.

Auxin signaling and transporters coordinate dynamic auxin gradients in leaf cells, fine‐tuning the acquisition of pavement‐cell shape and cell interdigitation.^[^ ^27^, ^28^ ^]^ To further explore whether auxin transport is involved in nitrate‐stimulated leaf growth, we used a jigsaw‐puzzle‐like cell‐shape model in leaf epidermal cells. We simultaneously treated hydroponically grown soybean with high nitrate in roots and NPA in leaves. Compared to high‐nitrate treatment alone, NPA addition attenuated the promotive effect of high nitrate on leaf area (Figure 1E; Figure S1B, Supporting Information), indicating that nitrate‐mediated leaf expansion requires functional auxin transport.

### 2.2 Soybean PIN3a and PIN3b Control Directional Auxin Transport

Twenty‐three *PINs* have been identified in the soybean genome, comprising *PIN1a‐e*, *PIN2a–b*, *PIN3a–d*, *PIN5a–b*, *PIN6a–b*, *PIN8a–d* and *PIN9a–d* (Figure S1C, Supporting Information).^[^ ^29^, ^30^ ^]^ Among them, the canonical plasma membrane‐localized PINs are primarily responsible for directing the intercellular flow of auxin.^[^ ^31^ ^]^ To identify the key PIN/s involved in soybean leaf growth, we analyzed the expression of the canonical *PIN1a–e*, *PIN2a*, *b*, and *PIN3a–d* in both young and mature leaves. Among these, *PIN1a–d* and *PIN3a*, *b* were relatively highly expressed in young leaves (Figure 1F), consistent with Arabidopsis PIN1 involvement in lobe initiation and pavement‐cell interdigitation.^[^ ^32^ ^]^ As the leaves matured, *PIN1* expression decreased, with *PIN3a* and *PIN3b* remaining abundant (Figure 1F). To confirm the expression pattern of soybean *PINs*, we developed transgenic plants carrying *PIN_pro_:GUS* reporter constructs, whereby *GUS* was driven by a 2‐kb promoter region upstream of *PIN* translational start codons. In mature leaves, strong GUS histochemical signals were observed for *PIN3a_pro_* and *PIN3b_pro_* lines, while *PIN1a–e_pro_* lines had no detectable signal (Figure 1H; Figure S1D, Supporting Information). Strong *PIN3a*‐ and *PIN3b*‐driven GUS signals were in general agreement with qRT–PCR data.

Given the promotive effect of nitrate on leaf growth in both young and mature leaves, and prominent *PIN3* expression levels therein, we hypothesized that *PIN3a* and *PIN3b* play major roles in this process. To further elucidate their functionality, we first examined the subcellular localization and functional conservation of PIN3 orthologs across species. In Arabidopsis roots, AtPIN3 exhibits distinct subcellular localization patterns: it is basally localized in the stele, apolar in root‐cap columella cells, and laterally in the endodermis.^[^ ^33^ ^]^ In Arabidopsis leaves, AtPIN3 associates with the adjacent cell membrane, and its dynamic localization is crucial for lobe formation.^[^ ^28^ ^]^ This polar localization of PIN3 precisely regulates auxin transport and distribution, which is essential for proper organ development.^[^ ^24^ ^]^ Soybean has four *PIN3* orthologs (Figure S1C, Supporting Information), which are differentially expressed in leaves (Figure 1H). To assess functional conservation between GmPIN3 and AtPIN3, we fused GmPIN3s to GFP under the control of the *AtPIN3* promoter, wherein GFP was inserted in the hydrophilic loop of GmPIN3a–d at a position comparable to AtPIN3–GFP.^[^ ^34^ ^]^ These four constructs, designated *AtPIN3_pro_:GmPIN3*–*GFP*, were introduced into the Arabidopsis *pin3‐4* null mutant. All transgenic lines partially rescued the *pin3‐4* agravitropic‐hypocotyl mutant phenotype (Figure S1E,F, Supporting Information). GmPIN3–GFP fluorescence in the root stele was observed at the basal and lateral sides of the plasma membrane (Figure S1G, Supporting Information), consistent with AtPIN3 subcellular polarity.^[^ ^33^ ^]^ These experiments suggest that GmPIN3a–d exhibit functional conservation with AtPIN3.

Among all PIN3 homologues, *PIN3a* and *PIN3b* were the most highly expressed in leaves (Figure 1F), whereas *PIN3c* was undetectable. We therefore used CRISPR–Cas9 to generate *pin3a* and *pin3b* single mutants as well as a *pin3ab* double mutant. Because PIN3d also showed detectable transcript in leaves (Figure 1F), we additionally created two independent *pin3abd* triple mutants (designated *pin3abd^CR1^* and *pin3abd^CR2^*, Figures S2 and S3, Supporting Information). The single mutants did not show obvious phenotypic differences compared to WT, but the double and triple mutants had curlier and greener leaves than WT (Figure 1G). *pin3ab* and *pin3abd* mutants had statistically significantly higher chlorophyll contents (calculated as SPAD value) and a longer stay‐green stage compared to WT (Figure 1I; Figure S4A–C, Supporting Information). To investigate PIN3 auxin‐transport capacity, IAA efflux was assessed using microelectrode‐based non‐invasive micro‐test technology (NMT, Figure S4D, Supporting Information). IAA efflux rates in *pin3ab* and *pin3abd* mutants were lower than WT (Figure 1J). PIN3a and PIN3b, therefore, play major roles in controlling leaf growth in soybean.

### 2.3 PIN3a and PIN3b Negatively Regulate Nitrate Uptake Independently of Symbiotic Nitrogen Fixation

**Figure 2:**
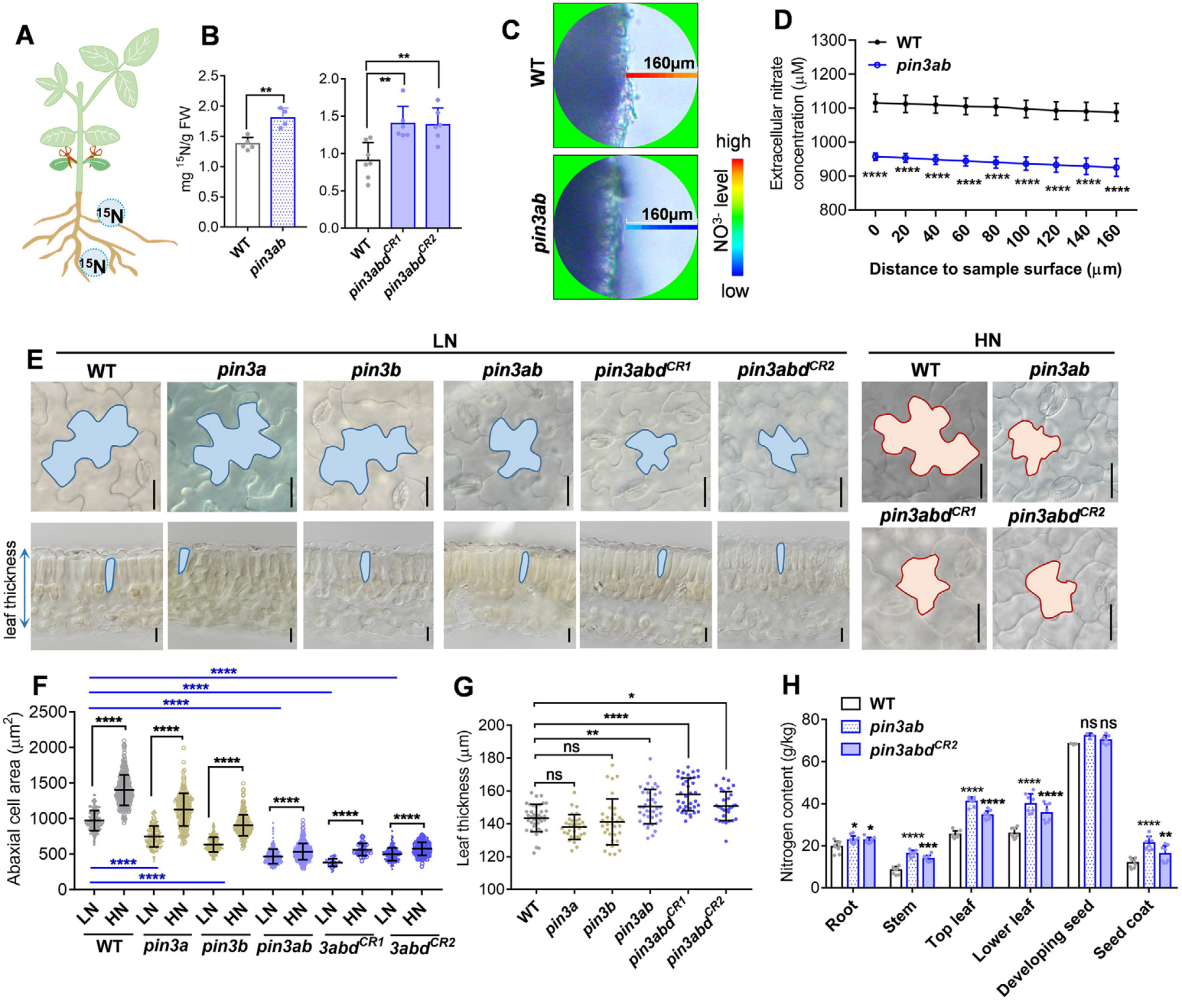
Soybean PIN3a and PIN3b are involved in the nitrate response for pavement‐cell growth. A,B) Summary of treatment scheme (A) and ^15^N quantification at leaf level (B). 14‐day‐old WT, *pin3ab* and *pin3abd^CR^* plants were incubated in 5.3 mm ^15^NO_3_^−^‐containing solution for 7 h, then all leaves were harvest for ^15^N detection. Data are the mean ± SD of *n* = 5 or 7 for WT, 4 for *pin3ab*, 6 for *pin3abd* samples. *p*‐values were determined by two‐tailed Student's *t*‐test (left) and one‐way ANOVA with Dunnett's test (right) (^**^ *p* < 0.01). C,D) A representative picture showing detection of concentration gradients of nitrate in pavement cell of 10‐day‐old WT and *pin3ab* mutant plants. Low to high concentrations of nitrate are indicated according to bar color. Pavement cell surface of* pin3ab *mutant exhibited decreased concentration of extracellular nitrate (D). Data are the mean ± SD of *n* = 9 (D) for each point, respectively. *p*‐values were determined by two‐way ANOVA Sidak's multiple comparisons test (^****^ *p* < 0.0001). E–G) Analysis of pavement cells in 6‐day‐old WT, *pin3ab* and *pin3abd^CR^ *plants transferred to high‐ or low‐nitrogen conditions for an additional 5 days. Leaf pavement cells were observed by light microscopy. Representative cells were individually trackedE) and abaxial cell area quantified (F). Leaves were sliced perpendicular to the direction of cell expansion (E, lower panels), and leaf thickness was quantified G). Data are the mean ± SD of *n* = 306, 297, 464, 450, 540, 482, 323, 367, 44, 40, 524, and 465 samples (F) and 40, 32, 33, 40, 37, and 31 samples (G) for each condition, respectively. Scale bar in (E) = 20 µm. *p*‐values were determined by two‐way ANOVA with Tukey's multiple‐comparisons test (F) and one‐way ANOVA with Dunnett's test (G) (^*^ *p* < 0.05; ^**^ *p* < 0.01; ^****^ *p* < 0.0001; ns, non‐significant). H) Nitrogen concentrations in different organs harvested from 55‐day‐old WT, *pin3ab* , and *pin3abd^CR2^* plants. Data are the mean ± SD of *n* = 8 samples. *p*‐values were determined by two‐way ANOVA with Tukey's multiple‐comparisons test (^*^ *p*< 0.05; ^**^ *p* < 0.01; ^***^ *p*< 0.001; ^****^ *p* < 0.0001; ns, non‐significant).

To further investigate whether PIN3 is involved in nitrate acquisition, we examined nitrate‐uptake capacity by applying ^15^NO_3_^−^to the roots of hydroponically grown plants for 7 h and subsequently measured ^15^N levels in leaves. The *pin3ab* and *pin3abd^CR^* mutants contained statistically significantly higher levels of ^15^N than WT (**Figure** **2**A,B). When ^15^NO_3_^−^ was supplied to the roots for 24 h and leaves were harvested from different nodes, all sampled *pin3ab* leaves had more ^15^N than WT, regardless of node position (Figure S5A,B, Supporting Information). Furthermore, we visualized nitrate uptake using NMT technology coupled with a real‐time imaging system. WT and *pin3ab* pavement leaves were balanced in a 1 mm potassium nitrate test solution for 40 min, after which the nitrate concentration was determined at various distances (0–160 µm, with 20‐µm intervals) from the sample surface. The extracellular nitrate concentration in WT plants was higher than in *pin3ab* mutants, indicating increased nitrate influx in *pin3ab* compared to WT (Figure 2C,D).

Deficiencies in PIN transporters, as observed in the Arabidopsis *pin3/4/7* mutant, disrupt pavement‐cell polarity and reduce cell lobing.^[^ ^28^ ^]^ To investigate the role of *PIN3a* and *PIN3b* in soybean responses to nitrate, we examined the nitrate‐stimulated pavement‐cell expansion. Under low‐nitrate conditions, the pavement‐cell area of *pin3a* and *pin3b* single mutants was significantly smaller than that of WT. Pavement cells in the double and triple mutants were even smaller and had fewer and shorter lobes compared to WT (Figure 2E,F; Figure S5C–E, Supporting Information). These lobing defects were consistent with the Arabidopsis *pin3/4/7* mutant.^[^ ^28^ ^]^ Transverse sectioning of leaves revealed that *pin3ab* and *pin3abd^CR^* mutants had thicker leaves than WT, while single mutants were unchanged (Figure 2E,G). Palisade‐cell length of *pin3ab* and *pin3abd* mutants was comparable to WT (Figure S5F, Supporting Information), suggesting the thicker leaves observed in *pin3ab* and *pin3abd^CR^* mutants are primarily due to the thicker spongy mesophyll on the abaxial side. The isotropic growth of *pin3ab* and *pin3abd^CR^* leaves in the anticlinal and periclinal axes further supports the importance of PIN3a and PIN3b in soybean leaf cell polarity formation.

In response to high nitrate levels, WT, *pin3a*, and *pin3b* single mutants exhibited a 42–51% increase in pavement‐cell area. In contrast, the area for *pin3ab* and *pin3abd^CR^* mutants only increased by 14–15% (Figure 2E,F). This reduced sensitivity of *pin3* higher‐order mutants to high nitrate implicates *PIN3a* and *PIN3b* in the nitrate regulation of leaf‐cell expansion.

The aforementioned experiments consistently demonstrate that *PIN3a* and *PIN3b* are required for nitrate acquisition, which prompted us to quantify nitrogen contents in source and sink organs. Especially in the above‐ground organs such as the stem, leaf, and seed coat, the *pin3ab* and *pin3abd^CR2^* mutants had statistically significantly higher nitrogen levels compared to WT plants (Figure 2H). This suggests that nitrate acquisition is globally up‐regulated in the sink organs of *pin3ab* and *pin3abd^CR2^* mutants.

Given that soybean primarily obtains its nitrogen through symbiotic nitrogen fixation, we investigated whether the elevated nitrogen content in the *pin3ab* and *pin3abd^CR2^* mutants results from enhanced nitrogen fixation. Following rhizobia inoculation, nodule numbers and nitrogen‐fixation capacity of *pin3ab* and *pin3abd^CR^* mutants were statistically lower in mutants than in WT under low nitrate conditions (Figure S5G–I, Supporting Information). High nitrate severely suppressed nodule formation in both WT and *pin3* mutants (Figure S5G,I, Supporting Information). Nevertheless, even under high‐nitrate/rhizobia‐co‐supplied conditions, *pin3ab* and *pin3abd^CR2^* leaves still accumulated higher nitrogen than WT (Figure S5J, Supporting Information). This finding indicates that the high nitrogen levels in the *pin3ab* and *pin3abd^CR^* mutants are not attributable to enhanced nodule symbiosis but rather to increased nitrate uptake.

### 2.4 High Nitrate Concentrates PIN3a to Cell‐Wall Junctions by Accelerating PIN3a Degradation

**Figure 3:**
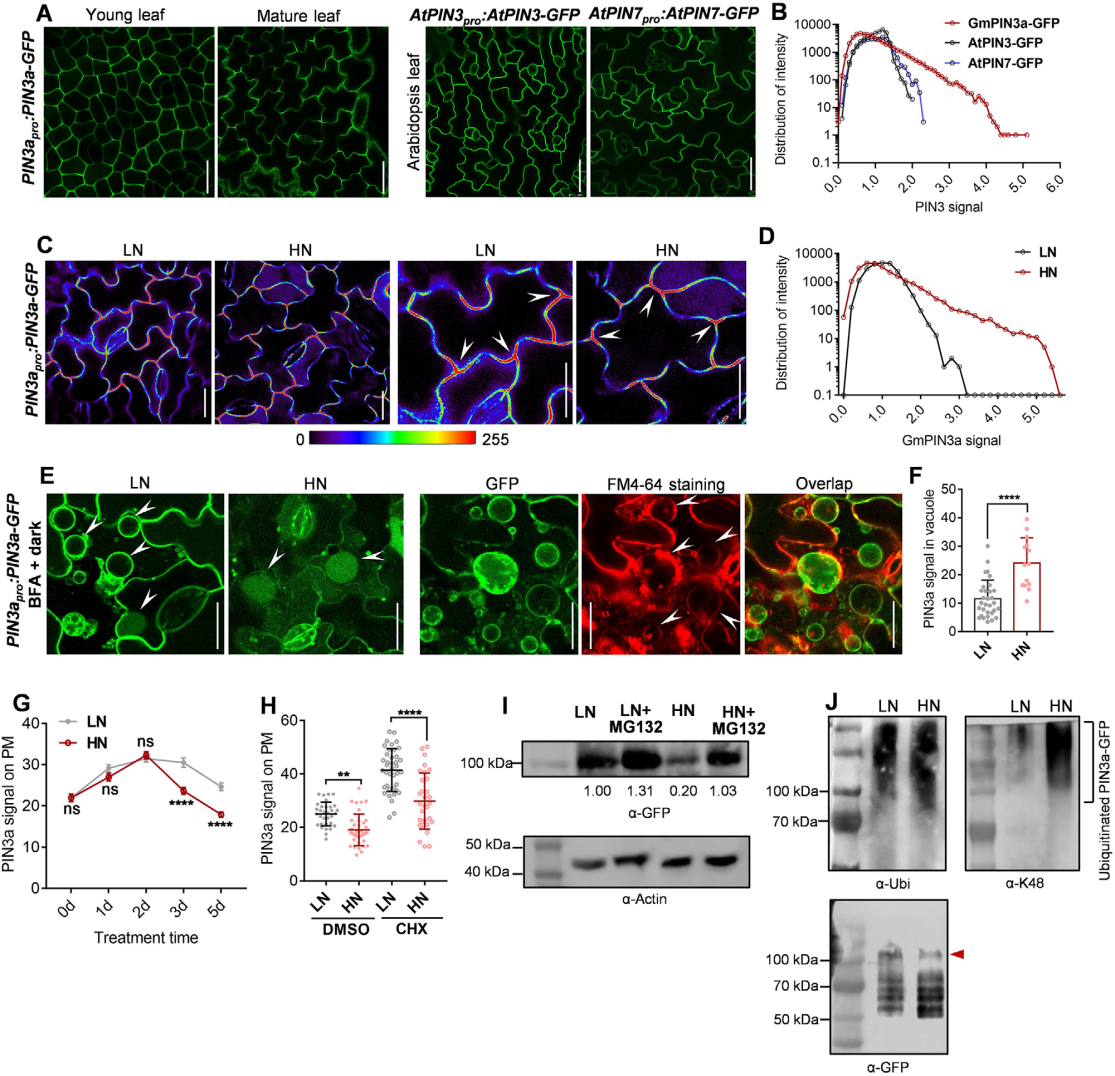
High nitrate targets PIN3a to cell junctions by accelerating PIN3a degradation. A, B) Confocal‐microscopy images of PIN3a–GFP subcellular localization in 5‐ and 11‐day‐old leaves on the abaxial surface from *PIN3a_pro_:PIN3a*–*GFP* stable‐transgenic soybean plants grown under low‐nitrogen conditions (A, left panels), and AtPIN3–GFP and AtPIN7–GFP subcellular localization in 5‐day‐old leaves from *AtPIN3_pro_:AtPIN3*–*GFP* and *AtPIN7_pro_:AtPIN7*–*GFP* transgenic Arabidopsis plants grown under low‐nitrogen conditions (A, right panels). Plasma‐membrane‐distributed GmPIN3a–GFP, AtPIN3–GFP , and AtPIN7–GFP were measured along the whole membrane and shown as discrete values (B, *n* = 49237, 41513 , and 30782 from 15 independent pictures; AU, arbitrary unit). Scale bar = 20 µm (A). C,D) Confocal‐microscopy images of PIN3a–GFP. 6‐day‐old *PIN3a_pro_:PIN3a*–*GFP* stable‐transgenic plants were transferred to low‐ or high‐nitrogen conditions for 5 days before imaging (C). Pictures with magnification were presented in the right panels (C). Plasma membrane‐associated GmPIN3a–GFP in low‐ or high‐nitrogen conditions was measured along the whole membrane and shown as discrete values (D, *n* = 21413 and 24433). GmPIN3a–GFP accumulating at cell–cell junctions is highlighted by arrows. Scale bar = 10 µm (C). Low to high signal of GmPIN3a–GFP is indicated according to bar color. E,F) Confocal microscopy of PIN3a–GFP. 6‐day‐old *PIN3a_pro_:PIN3a*–*GFP* stable‐transgenic soybean plants were transferred to low‐ or high‐nitrogen conditions for 3 days, then leaves were sprayed with 200 µm brefeldin A and incubated in darkness for 2 h (E, left panels). *PIN3a_pro_:PIN3a*–*GFP* leaves were co‐stained with FM4‐64, and GFP signal (green) was overlapped with FM4‐64‐indicated vacuolar signal (red) (E, right panels). PIN3a–GFP localization to vacuoles is highlighted by arrows (E). PIN3a signal within vacuoles was calculated in F. Data are the mean ± SD of *n* = 31 and 14 samples. *p*‐values were determined by a two‐tailed Student's *t*‐test (^****^ *p *< 0.0001). G) 6‐day‐old *PIN3a_pro_:PIN3a*–*GFP* stable‐transgenic plants were transferred to low‐ or high‐nitrogen conditions for an additional 1–5 days, membrane‐associated PIN3a–GFP signal was quantified by Image J. Data are the mean ± SD of n > 43 cells for each time point. *p*‐values were determined by two‐way Tukey's multiple comparisons test (^****^ *p* < 0.0001; ns, non‐significant). H) 6‐day‐old *PIN3a_pro_:PIN3a*–*GFP* plants were transferred to low‐ or high‐nitrogen conditions for an additional 5 days, and treated with 100 µm cycloheximide (CHX) for 6 h. Membrane‐associated PIN3a–GFP signal was quantified by Image J. Data are the mean ± SD of *n* = 31–39 samples. *p*‐values were determined by two‐way Tukey's multiple comparisons test (^**^ *p*< 0.01; ^****^ *p* < 0.0001). I,J) Western blots from 6‐day‐old *PIN3a_pro_:PIN3a*–*GFP* transgenic plants moved to low‐ or high‐nitrogen conditions for an additional 5 days before 20 µm MG132 application to leaves for 6 h before harvest. PIN3a–GFP abundance was detected with anti‐GFP antibody (I). Actin was used as the loading control (anti‐Actin, I). GFP‐tagged PIN3a was immunoprecipitated by GFP magnetic beads. The immunoprecipitated PIN3a proteins were detected by anti‐ubiquitin (α‐Ub) and anti‐K48‐linkage‐specific polyubiquitin (α‐K48) antibodies (J). PIN3‐GFP level was used as the loading control (anti‐GFP, J). Three biological replicates were performed.

Data so far suggest *PIN3a/b* negatively regulates soybean nitrate transport. We therefore sought to further understand how *PIN3a/b* respond to high‐nitrate levels. Considering the functional conservation and consistent polarization of both PIN3a–GFP and PIN3b–GFP in transgenic Arabidopsis roots, we selected PIN3a as a representative for studying polarity dynamics in leaf development. Functionality of a *PIN3a_pro_:PIN3a–GFP* construct in transgenic soybean plants in WT and *pin3ab* backgrounds was evidenced by restoration of pavement‐cell area and lobing defects in the *pin3ab* mutant (Figure S6A–E, Supporting Information). PIN3a–GFP predominantly localized to the plasma membrane in both leaf vasculature and pavement cells (**Figure** **3**A; Figure S6F, Supporting Information).

Prior to lobe formation in pavement cells of young leaves, PIN3a–GFP was distributed fairly uniformly on the plasma membrane, with a stronger signal observed at cell–cell junctions (Figure 3A). However, in mature leaves, wherein lobes are fully formed, PIN3a–GFP localization was less uniform and exhibited a striking preferential accumulation instead at tripartite cell‐wall junctions (Figure 3A). This change in PIN3a–GFP localization in pavement cells implicates PIN3a in soybean leaf development.

The distinct GmPIN3a–GFP polarization pattern differs from AtPIN3 and AtPIN7, which displayed a more uniform distribution on the plasma membrane of pavement cells in transgenic Arabidopsis (Figure 3A). To quantify polarization patterns, we compared the surface distribution of PIN3a–GFP by measuring discrete values of membrane‐associated GFP signal, with a higher discrete value indicating a more concentrated localization of PIN3a–GFP at cell junctions. GmPIN3a–GFP indeed displayed a wider range of signal distribution compared to AtPIN3–GFP and AtPIN7–GFP (Figure 3B). Under high nitrate, GmPIN3a–GFP was further enriched at cell junctions (Figure 3C,D).

Toward understanding how high nitrate enriches PIN3a–GFP at cell junctions, we wondered whether PIN3a–GFP dynamics are influenced by either lateral diffusion on the plasma membrane or endocytic internalization/recycling perpendicular to the plasma membrane. To investigate these hypotheses, we employed variable‐angle total internal‐reflection fluorescence microscopy (VA–TIRFM) to image cell‐surface‐associated PIN3a–GFP at the nanoscale and study its lateral dynamics at the single‐particle level. High nitrate did not appear to affect the velocity of lateral diffusion of surface‐associated PIN3a–GFP, at least under this setup (Figure S6G–I, Supporting Information), suggesting that high nitrate was unable to relocalize PIN3a to cell junctions.

PIN proteins undergo constitutive endocytic cycling/recycling, either to different domains of the membrane or for rerouting to the lytic vacuole for degradation.^[^ ^35^ ^]^ Proteolytic turnover of PINs requires their ubiquitination either mono‐ubiquitination or K63‐linked polyubiquitin chains, ultimately resulting in their vacuolar targeting.^[^ ^36^, ^37^ ^]^ To examine how high nitrate affects the PIN3a–GFP internalization, we treated leaves with the exocytosis inhibitor brefeldin A (BFA), which leads to the accumulation of endocytic proteins in endosomal compartments.^[^ ^38^ ^]^ Compared to low nitrate, high nitrate increased the cytosolic PIN3a–GFP signal in BFA‐induced compartments and lytic vacuoles while depleting PIN3a–GFP from the plasma membrane (Figure 3E,F). PIN3a–GFP accumulation in lytic vacuoles implies that high nitrate accelerates PIN3a degradation.

To confirm whether high nitrate promotes PIN3a degradation, we tracked plasma membrane‐associated PIN3a–GFP signal over 5 d under low‐ and high‐nitrate conditions. Prolonged exposure to high nitrate statistically significantly reduced PIN3a–GFP abundance, in comparison to low‐nitrate conditions (Figure 3G). We then treated leaves with cycloheximide (CHX) to inhibit protein synthesis. In the presence of CHX, PIN3a–GFP abundance on the plasma‐membrane was still compromised by high‐nitrate conditions (Figure 3H; Figure S6J, Supporting Information). Despite the identity of the machinery involved in proteasome‐mediated PIN degradation being unclear, treatment with 26S‐proteasome inhibitor MG132 stabilizes PINs.^[^ ^36^ ^]^ To determine whether a similar mechanism is at play in soybean, we observed a decrease in PIN3a–GFP abundance by anti‐GFP western blotting in leaves from the high‐nitrate condition compared to the low‐nitrate. MG132 addition inhibited nitrate‐induced PIN3a–GFP degradation (Figure 3I).

To determine whether PIN3 degradation occurs as a result of ubiquitination, we used anti‐ubiquitin (Ub) and anti‐K48‐linked polyubiquitin antibodies to assay proteasomal degradation of PIN3a–GFP. Stronger smeared bands indicative of PIN3a–GFP ubiquitination were observed in high‐nitrate‐treated leaves compared to the low‐nitrate condition (Figure 3J). In summary, microscopy and protein‐detection techniques provide evidence that high nitrate accelerates the degradation of PIN3a–GFP.

### 2.5 PIN3a and PIN3b Negatively Regulate NRT‐Dependent Nitrate Transport

**Figure 4:**
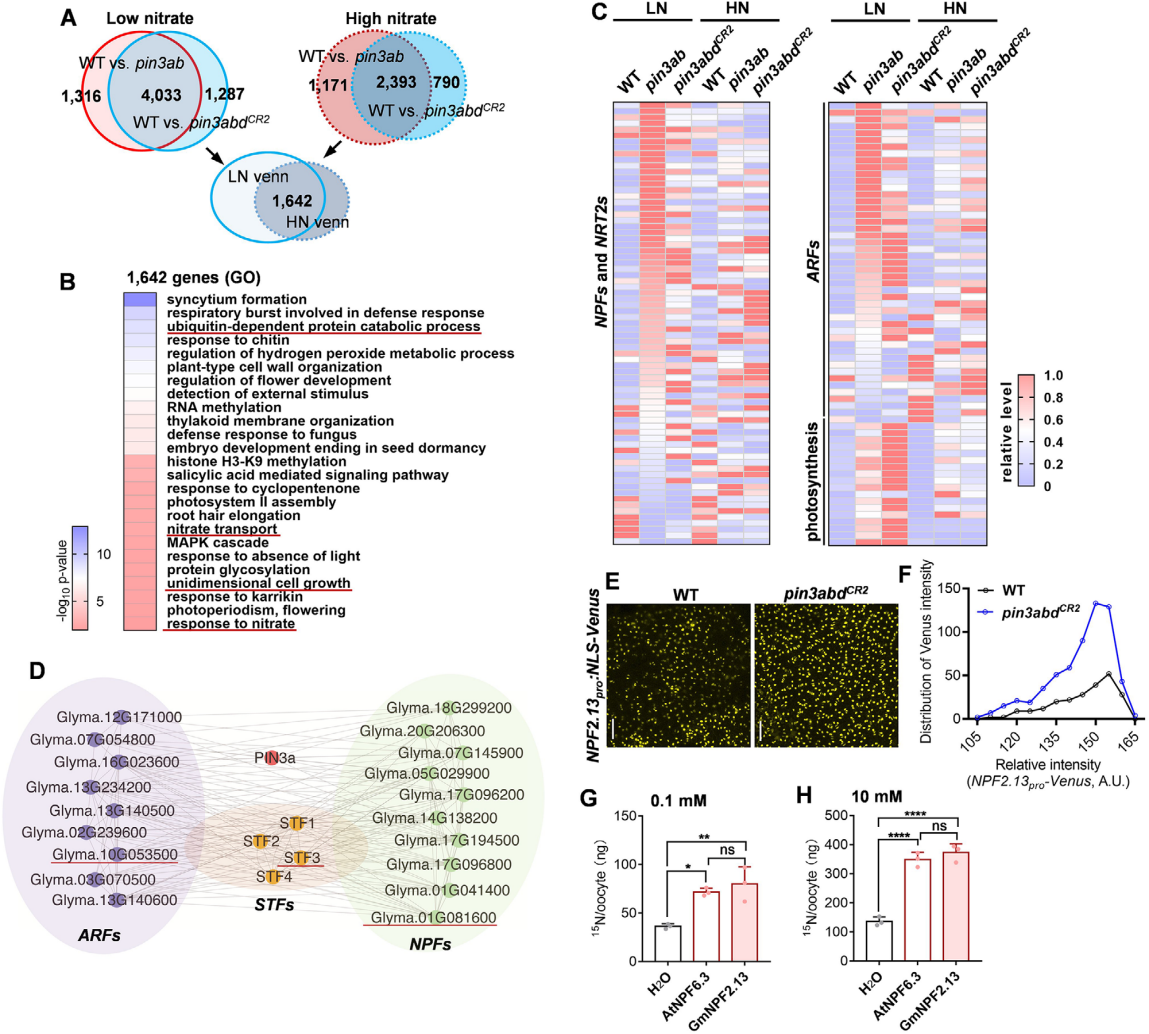
Transcriptome analysis of soybean *pin3* mutants. A) Venn diagram of DEGs identified in 6‐day‐old WT, *pin3ab*, and *pin3abd^CR2^* mutants transferred to low‐nitrate or high‐nitrate conditions for an additional 5 days before harvest. B) GO‐category classification of the 1642 co‐regulated genes as identified in (A). C) Heatmap depicting relative expression of *NITRATE TRANSPORTER 1 (NRT1)/PEPTIDE TRANSPORTER (PTR)* families (*NPFs*) and *NITRATE TRANSPORTER2S* (*NRT2s*), *AUXIN‐RESPONSE FACTORS* (*ARFs*), photosynthesis‐related genes after transfer to high‐ or low‐nitrate conditions. D) Co‐expression network for *PIN3a*. *STF3*, *ARF10*, and *NPF2.13 are* indicated by a red underline. E,F) *NPF2.13* promoter activity in transgenic soybean leaves expressing a *NPF2.13_pro_:NLS–Venus* reporter in WT and *pin3abd^CR2^* backgrounds visualized by confocal microscopy (E). The Venus signal was measured in each nuclear discrete values (F, *n* = 223 and 608, relative fluorescence intensity of each nuclear discrete in the *x*‐axis), and the distribution frequency is shown on the *y*‐axis (F). Scale bar = 10 µm. G,H) Nitrate‐transport activity of NPF2.13 in *Xenopus* oocytes. Oocytes were injected with cRNAs encoding *AtNPF6.3* or *GmNPF2.13* or the H_2_O negative control, then incubated with 0.1 mmol (G) or 10 mmol ^15^NO_3_^−^ (H) and monitored by continuous‐flow mass spectrometry. Data are the mean ± SD of *n* = 3 independent replicates for each point, *n* = 4–8 eggs for each replicate. *p*‐values were determined by one‐way ANOVA with Dunnett's multiple‐comparisons test (^*^ *p* < 0.05; ^**^ *p* < 0.01; ^****^ *p* < 0.0001; ns, non‐significant).

To understand why nitrate accumulates in *pin3ab* and *pin3abd^CR^* mutants, we performed RNA‐seq on leaves from WT, *pin3ab*, and *pin3abd^CR2^* plants grown under low‐ and high‐nitrate conditions. We applied a statistical significance threshold of *p*< 0.01 and a log_2_ fold‐change ≧ 2 to identify differentially expressed genes (DEGs). Compared to WT, 1642 genes were co‐regulated in *pin3ab* and *pin3abd^CR2^* leaves under both nitrate regimes (**Figure** **4**A; Table S1, Supporting Information). Gene‐ontology (GO) enrichment analysis revealed that “ubiquitin‐dependent protein‐catabolic process” was over‐represented (Figure 4B; Table S2, Supporting Information), consistent with observations of high nitrate promoting PIN3a degradation. Additionally, “unidimensional cellular‐growth event” was over‐represented in the mutants, which aligns with the role of *PIN3a/b* in abaxial–adaxial leaf development (Figure 4B; Table S2, Supporting Information).

Enriched GO terms also included “nitrate transport” and “nitrate‐response pathways,” and *NPF* transcripts were up‐regulated in *pin3ab* and *pin3abd^CR2^* mutants (Figure 4C; Table S3, Supporting Information). In contrast, genes encoding enzymes involved in nitrate metabolism, such as *GLUTAMINE SYNTHETASE* (*GS*) and *ASPARAGINE SYNTHETASE* (*AS*) were down‐regulated (Figure S7A and Table S3, Supporting Information). Glutamine synthetase catalyzes glutamine biosynthesis by incorporating a molecule of ammonium into glutamate, and asparagine synthetase transfers the glutamine or NH_4_^+^ amide group to aspartate to form asparagine and glutamate.^[^ ^4^ ^]^

Transcriptome data indicated that *PIN3* deficiency led to induction of nitrate transport but repression of nitrate metabolism, and weighted gene correlation network analysis (WGCNA) indicated co‐expression of *NPFs* and *PIN3a* (Figure 4D; Figure S7B and Table S4, Supporting Information). Within this module, *NPF7.3* (*Glyma.20g206300*) and *NPF2.13* (*Glyma.01g081600*) ranked highest both in overall module significance and in leaf‐specific transcript abundance (Table S3, Supporting Information). RT–qPCR confirmed elevated transcript levels of *NPF7.3* and *NPF2.13* in *pin3ab* and *pin3abd^CR2^* leaves compared with WT (Figure S7C, Supporting Information). We therefore generated constructs expressing nuclear‐localization signals (NLS) fused to Venus under the control of *NPF2.13* and *NPF7.3* promoters (*NPF_pro_:NLS–Venus*). These constructs were introduced into both WT and *pin3abd^CR2^* plants. Venus fluorescence was much brighter in *pin3abd^CR2^* compared to WT, indicating stronger expression of these *NPF* genes in *pin3abd^CR2^* mutants (Figure 4E,F; Figure S7D,E. Supporting Information). We then examined NPF2.13 nitrate‐uptake capacity in *Xenopus* oocytes*. NPF2.13* complementary RNAs (cRNAs) were injected into oocytes, and after 2 d of incubation with either 0.1 or 10 mm ^15^NO_3_^−^, isotope abundance was measured. Oocytes injected with *NPF2.13* had a high nitrate‐transport ability, comparable to that of Arabidopsis *NPF6.3* (Figure 4G,H). Therefore, the enhanced nitrate uptake in *pin3ab* and *pin3abd^CR^* mutants is likely due to the transcriptional up‐regulation of functional nitrate transporters.

### 2.6 An ARF10–NPF2.13 Module Promotes Nitrate Uptake in Higher‐Order *Pin3* Mutants

**Figure 5:**
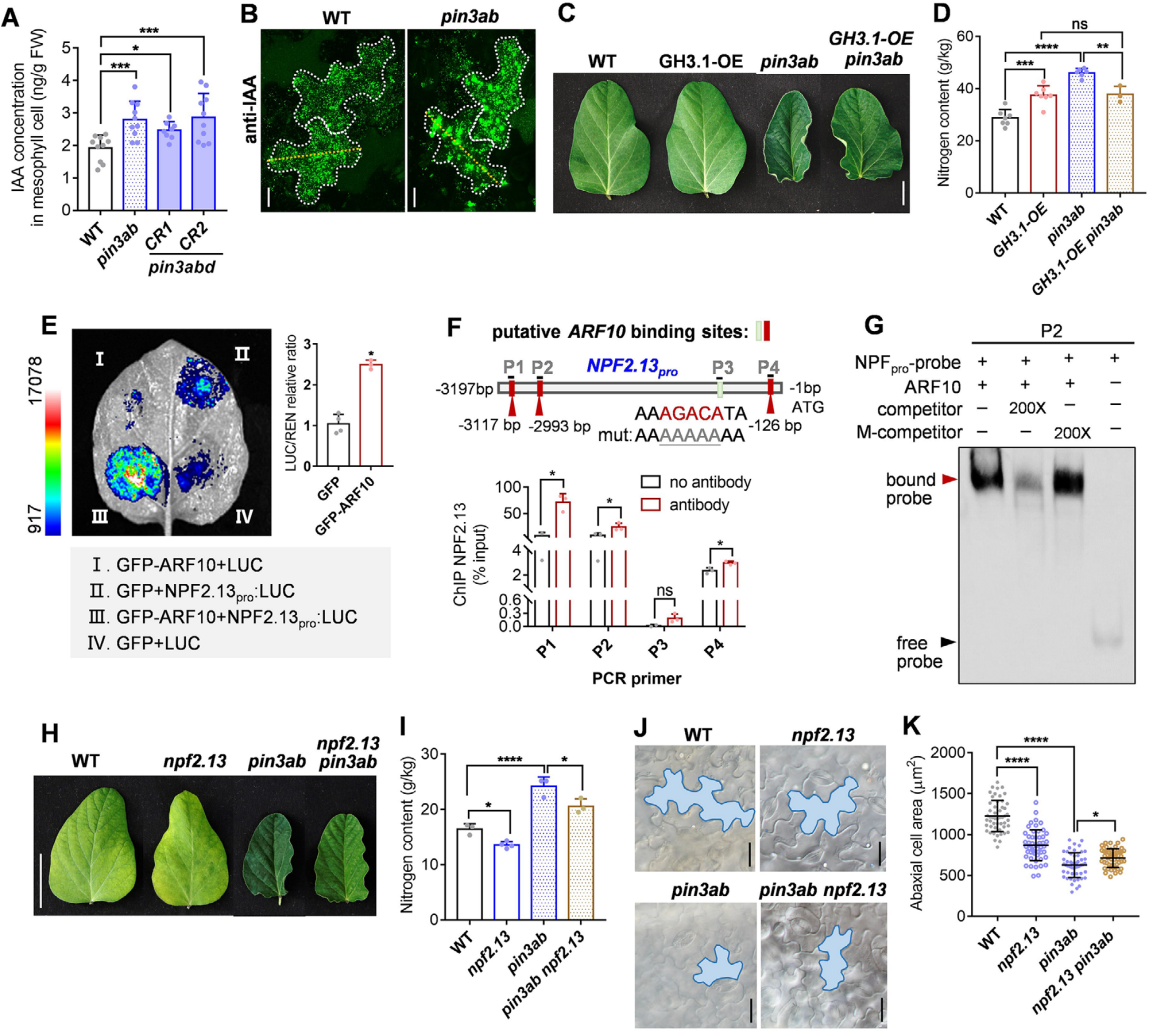
ARF10 activates *NPF2.13* expression to promote nitrate uptake in *pin3ab* mutants. A) Quantification of IAA in mesophyll cells and pavement cells by UPLC in 10‐day‐old WT, *pin3ab*, and *pin3abd^CR^* mutants. Data are the mean ± SD of *n* = 6 or 7 for each genotype. *p*‐values were determined by one‐way ANOVA with Fisher's LSD test (^*^ *p* < 0.05; ^***^ *p* < 0.001). B) Anti‐IAA antibody immunostaining of auxin distribution in leaf pavement cells of 11‐d‐old WT and *pin3ab* plants. Representative cell shapes are indicated by white dotted lines. The IAA signal was tracked along the indicated orange lines (see Figure S8B, Supporting Information). Scale bar = 10 µm. C,D) Characterization of transgenic plants of over‐expressing *GH3.1* (*GH3.1‐OE*) in the *pin3ab* mutant background. Leaf morphology (C), nitrogen content (D) are shown. Data are the mean ± SD of *n* = 7, 7, 6, 3 samples for (D) for each point, respectively, Scale bar = 1 cm (C). *p*‐values were determined by one‐way ANOVA with Tukey's multiple‐comparisons test (^**^ *p* < 0.01; ^***^ *p*< 0.001; ^****^ *p* < 0.0001; ns, non‐significant). E) Dual‐luciferase reporter assay of ARF10 activating the *NPF2.13* promoter. Left: *N. benthamiana* leaf co‐infiltrated with different combinations (listed below) of reporter constructs and the transcription‐factor effector strains. Right: LUC/REN ratios. Data are the mean ± SD of *n* = 4, 3. *p*‐values were determined by Mann–Whitney test (^*^ *p* < 0.05). F) ChIP–qPCR of GFP–ARF10 association with the *NPF2.13* promoter. Top: Schematic of the 3.2‐kb *NPF2.13* promoter region with P1–P4 representing regions containing putative ARF10 binding sites (AGACA) used for amplification. Bottom: ChIP–qPCR assay of GFP–ARF10 enrichment. Data are the mean ± SD of *n* = 3. “No antibody” represents the mock‐IP negative control. *p*‐values were determined by two‐tailed Student's *t*‐test (^*^ *p* < 0.05; ns, non‐significant). G) EMSA of 6xHis–ARF10 directly binds the TGTC motif of the *NPF2.13* promoter in vitro. Unlabeled DNA probe in 200‐fold molar excess relative to the labeled probe was assayed, and competition with an unlabeled, mutated (M) competitor in 200‐fold excess was analyzed. P2: The 2978–3012‐bp region relative to the *NPF2.13* translational start codon, as shown in (F). H,I) Characterization of transgenic plants of WT, *npf2.13*, *pin3ab*, and *pin3ab npf2.13* mutants. Leaf morphology (H), nitrogen content (I) are shown. Data are the mean ± SD of *n* = 3, 4, 3, 3 samples for (I) for each point, respectively, Scale bar = 1 cm (H). *p*‐values were determined by one‐way ANOVA with Tukey's multiple‐comparisons test (^*^ *p* < 0.05; ^****^ *p* < 0.0001). J,K) Analysis of pavement cells in WT, *npf2.13*, *pin3ab*, and *pin3ab npf2.13* mutants. Leaf pavement cells were observed by light microscopy. Representative cells were individually tracked (J) and abaxial cell area quantified (K). Data are the mean ± SD of *n* = 51, 51, 52, and 51samples for (K) for each point, respectively. Scale bar in (J) = 20 µm. *p*‐values were determined by one‐way ANOVA with Tukey's test (K) (^*^ *p* < 0.05; ^****^p < 0.0001).

Knocking out PIN3 transporters is expected to result in over‐accumulation of auxin within cells due to impaired efflux. IAA quantification specifically in the vasculature and mesophyll confirmed over‐accumulation in *pin3ab* and *pin3abd* mesophyll cells compared to WT (**Figure** **5**A; Figure S8A, Supporting Information). Anti‐IAA immunostaining confirmed a brighter and more aggregated auxin signal in *pin3ab* pavement cells compared to WT (Figure 5B; Figure S8B, Supporting Information).

To validate whether excess auxin causes leaf defects in *pin3ab* and *pin3abd* mutants, we overexpressed *GRETCHEN HAGEN 3* (*GH3*) in the *pin3ab* mutant background. GH3 converts IAA to non‐physiologically active IAA–amino‐acid.^[^ ^39^ ^]^ Over‐expression of *GH3.1* (*GH3.1‐OE*) restored the curly leaves and defective pavement‐cell‐lobing phenotype of *pin3ab* mutants, bringing their leaf morphology closer to that of WT (Figure 5C; Figure S8C–E, Supporting Information). More importantly, high nitrogen content in *pin3ab* leaves was also partially restored in *GH3.1‐OE pin3ab* plants (Figure 5D), confirming a role for auxin for nitrate acquisition.

Transcripts encoding the core transcription factors *AUXIN RESPONSE FACTOR* (*ARF*) were up‐regulated in *pin3ab* and *pin3abd^CR2^* mutants (Figure 4C; Table S3, Supporting Information). WGCNA analysis also revealed co‐expression of *NPFs*, *ARFs*, and *PIN3a* (Figure 4D), suggesting a potential relationship in nitrate uptake. ARFs transmit auxin signals by binding to TGTC‐containing *cis*‐elements in target genes.^[^ ^40^ ^]^ TGTC(TC) elements are highly abundant in the promoters of soybean *NPFs* (Figure S8F, Supporting Information), suggesting that *ARFs* might activate *NPF* transcription. To check this, we selected leaf‐abundant *ARF10* from the *PIN3a*–*ARF*–*NPF* co‐expression module as a representative *ARF* gene. Transgenic plants expressing the *ARF10* promoter:GUS fusion (*ARF10_pro_:GUS*) showed GUS activity in leaf pavement cells (Figure S8G, Supporting Information), confirming *ARF10* expression in this tissue. Dual‐luciferase reporter assays and ChIP–qPCR showed that ARF10–GFP binds to and activates the *NPF2.13* promoter at the P1, P2, and P4 regions (Figure 5E,F). EMSA was used to determine whether ARF10 recognizes TGTC(TC) motifs in the *NPF2.13* promoter. Labeled probes carrying AGACAT motifs (complementary sequence: ATGTCT) were incubated with 6xHis–ARF10. Formation of 6xHis–ARF10–*NPF2.13_pro_* complexes was detected, and the complex was weakened upon co‐incubation with unlabeled probe competitors, but remained unchanged when mutated competitors were added (Figure 5G). Thus, ARF10 directly binds to the *NPF2.13* promoter to induce its transcription and trigger nitrate uptake.

We then knocked‐out *NPF2.13* in the WT or *pin3ab* mutant background to generate *npf2.13* and *npf2.13 pin3ab* mutants (Figures S9 and S10, Supporting Information). The high nitrogen content of *pin3ab* was compromised in *pin3ab npf2.13* triple mutant (Figure 5H,I). Moreover, *pin3ab* leaf‐cell defects were partially restored when *NPF2.13* was simultaneously disrupted (Figure 5J,K). These genetic analyses provide further evidence supporting the involvement of *PIN3a/b* in NPF2.13‐mediated nitrate acquisition.

### 2.7 ARF10–STF3 and STF3–NPF2.13 Genetic Modules Enhance Nitrate Uptake in *P* *in3* Multiple Mutants

The *PIN3a*‐responsive transcriptome also included the light‐signaling transcription factor *SOYBEAN TGACG‐MOTIF‐BINDING FACTOR* (*STF*) genes, which are co‐expressed with *ARFs* and *NPFs* (Figure 4D). Due to nitrogen over‐accumulation in *pin3ab* and *pin3abd^CR^* mutants, their leaves are greener and have enhanced photosynthetic efficiency (Figure S11A,B, Supporting Information). STF3/4 and the Arabidopsis homolog HY5 coordinate light signaling.^[^ ^41^ ^]^ qRT–PCR confirmed a five‐fold up‐regulation of *STF3* and a two‐fold up‐regulation of *STF4* in *pin3ab* and *pin3abd^CR2^* leaves (Figure S11C, Supporting Information), suggesting a potential role for STF3/4 in PIN3‐dependent nitrate uptake. *STF3/4* expression was also induced in WT plants by exogenous NAA treatment (Figure S11D, Supporting Information), mimicking the auxin over‐accumulation status in *pin3ab* and *pin3abd^CR^* mutants.

**Figure 6:**
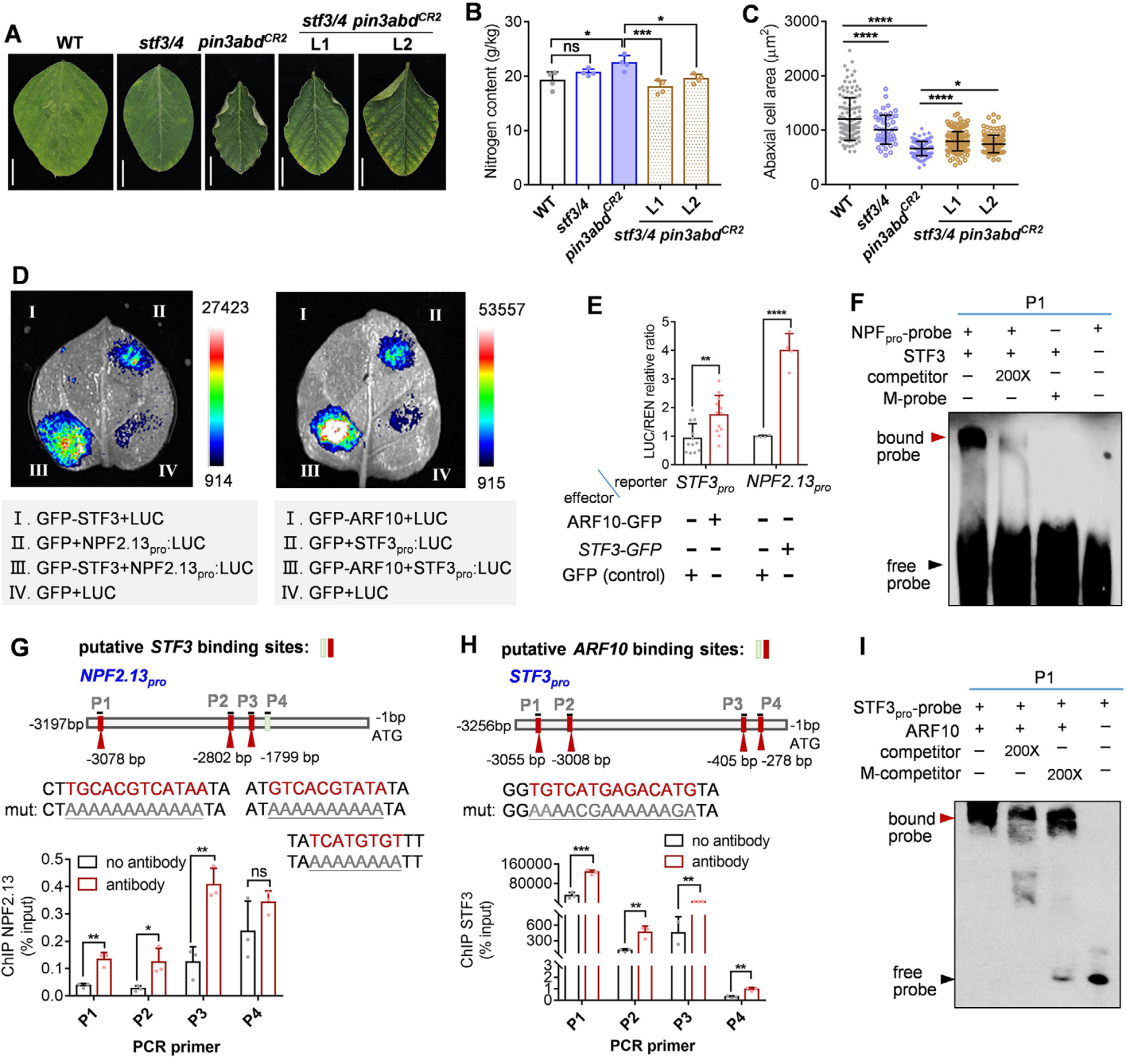
ARF10 activates STF3 and STF3 activates NPF2.13 expression to amplify nitrate uptake in pin3ab and pin3abd mutants. A,B) Characterization of WT, *stf3/4*, *pin3abd^CR2^* and two independent *stf3/4 pin3abd^CR2^* quintuple mutants. Leaf morphology (A), nitrogen content (B) are shown. Data are the mean ± SD of *n* = 4 samples for (B) for each point, respectively. Scale bar = 1 cm (A) *p*‐values were determined by one‐way ANOVA with Tukey's multiple‐comparisons test (^*^ *p* < 0.05; ^***^ *p* < 0.001; ns, non‐significant) C) Analysis of pavement cells in WT, *stf3/4*, *pin3abd^CR2,^* and two independent *stf3/4 pin3abd^CR2^* quintuple mutants. Leaf pavement cells were observed by light microscopy. Abaxial cell area quantified. Data are the mean ± SD of *n* = 129, 52, 138, 165, and 126 samples for each point, respectively. *p*‐values were determined by one‐way ANOVA with Tukey's multiple‐comparisons test (^*^ *p* < 0.05; ^****^ *p* < 0.0001). D,E) Dual‐luciferase reporter assays of STF3 activating the *NPF2.13* promoter (left) and ARF10 activating the *STF3* promoter (middle). Right: LUC/REN ratios. Data are the mean ± SD of *n* = 12, 12, 4, 4 samples from left to right. *p*‐values were determined by two‐tailed Student's t‐test (^**^ *p* < 0.01; ^****^ *p* < 0.0001). F) EMSA of 6xHis–STF3 directly binding to the G‐box motif (P1: 3063 to 3,104 bp relative to the translational start codon as shown in (G) present in the *NPF2.13* promoter in vitro. Unlabeled DNA probe in 200‐fold molar excess relative to the labeled probe was assayed, and competition with an unlabeled, mutated (M) probe in one‐fold excess was analyzed. G) ChIP–qPCR of STF3–GFP association with the *NPF2.13* promoter. Top: Schematic of the 3.2‐kb *NPF2.13* promoter region with P1–P4 representing regions containing putative STF3 binding sites (G boxes) used for amplification. Bottom: ChIP–qPCR assay of STF3–GFP enrichment. Data are the mean ± SD of *n* = 3. “No antibody” represents the mock‐IP negative control. *p*‐values were determined by two‐tailed Student's *t*‐test (^*^ *p* < 0.05; ^**^ *p* < 0.01; ns, non‐significant). H) ChIP–qPCR of GFP–ARF10 association with the *STF3* promoter. Top: Schematic of the 3.2‐kb *STF3* promoter region with P1–P4 representing regions containing putative ARF10 binding sites (AGACA, complementary sequence is TGTCT) used for amplification. Bottom: ChIP–qPCR assay of GFP–ARF10 enrichment. Data are the mean ± SD of *n* = 3. “No antibody” represents the mock‐IP negative control. *p*‐values were determined by two‐tailed Student's *t*‐test (^**^ *p* < 0.01; ^***^ *p* < 0.001). I) EMSA of 6xHis–ARF10 directly binding the TGTCTC motif of the *STF3* promoter in vitro. Unlabeled DNA probe in 200‐fold molar excess relative to the labeled probe was assayed, and competition with an unlabeled, mutated (M) competitor in 200‐fold excess was analyzed. P1: The 3040–3075‐bp region relative to the *STF3* translational start codon, as shown in (H).

STF3–GFP was strongly expressed in leaf pavement cells in transgenic *STF3_pro_:STF3–GFP* soybean plants (Figure S11E, Supporting Information). To clarify the involvement of STF3/4 in PIN3‐dependent nitrate acquisition, we knocked‐out *STF3* and *STF4* in WT and *pin3abd^CR2^* mutant backgrounds by CRISPR–Cas9 (Figures S11F,G and S12A,B, Supporting Information). Examination of leaf phenotype in *stf3/4 pin3abd^CR2^* quintuple mutants indicated that nitrogen content and leaf‐cell area of the *stf3/4 pin3abd^CR2^* quintuple mutants were partially restored compared to *pin3abd^CR2^* (**Figure** **6**A–C; Figure S11H, Supporting Information). This confirms that signaling of light cues via STF3 and STF4 contributes to enhancing nitrate acquisition at least in the *pin3abd^CR2^* mutant.

Arabidopsis HY5 binds to a light‐responsive C/G‐box (CACGTC) *cis*‐element in the *NRT2.1* promoter to enhance nitrate uptake.^[^ ^42^ ^]^ To determine whether STF3 acts upstream of *NPF2.13* in soybean, we investigated if STF3 binds its promoter. Dual‐luciferase reporter assays revealed that STF3 activated *NPF2.13* expression (Figure 6D,E), and EMSA with probes from the *NPF2.13* promoter carrying CACGTC motifs demonstrated the formation of STF3–*NPF2.13_pro_* complexes (Figure 6F; Figure S12C, Supporting Information). ChIP–qPCR with an anti‐GFP antibody confirmed recruitment of STF3–GFP to the P1–3 regions of the *NPF2.13* promoter in transgenic soybean leaves (Figure 6G). Similar approaches were employed to confirm ARF10 recruitment to the *STF3* promoter. ARF10–GFP could bind the *STF3* promoter and activate its expression (Figure 6D,E,H,I). These findings collectively demonstrate that *ARF10–STF3* and *STF3–NPF2.13* modules are responsible for enhanced nitrate uptake in *pin3ab* and *pin3abd^CR^* mutants.

### 2.8 Field‐Grown Pin3ab Mutants are Efficient at Nitrate Acquisition and have Improved Seed Oil

**Figure 7:**
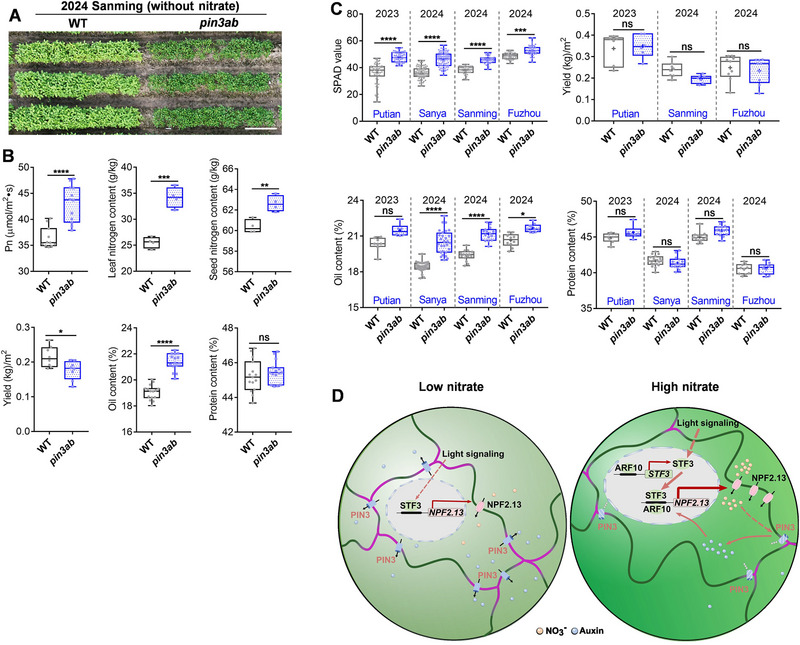
Field‐grown pin3ab double mutants contain more oil without a yield penalty. A) Aerial view of 50‐d‐old WT and *pin3ab* double mutants cultivated in Sanming in 2024, without nitrate fertilizer. Scale bar = 1 m. B) Quantification of photosynthetic rate in 45‐d‐old plants (Pn, *n* = 11 and 12), nitrogen level in leaves and seeds in 78‐d‐old plants (*n* = 4), yield per square meter (*n* = 8 and 9), seed‐oil and seed‐protein content (*n* = 15) for plants grown in Sanming in 2024 without nitrate fertilizer. *p*‐values were determined by two‐tailed Student's t‐test for Pn, nitrogen level, and yield, and were determined by the Wilcoxon test for oil and protein level (^*^ *p* < 0.05; ^**^ *p* < 0.01; ^***^p < 0.0001; ^****^ *p* < 0.0001; ns, non‐significant). C) SPAD value, yield per square meter, seed oil and protein contents in WT and *pin3ab* double mutants grown in Putian (2023), Sanya (2024), Sanming (2024) and Fuzhou (2024) fields. Data are the mean ± SD of *n* = 54, 58, 90, 88, 45, 45, 45, 45 samples (for SPAD), *n* = 6, 6, 8, 7, 9, 9 samples (for yield), *n* = 8, 6, 30, 28, 15, 15, 10, 10 (for oil) and *n* = 8, 6, 18, 15, 13, 14, 10, 10 (for protein) detection, from left to right. *p*‐values were determined by one‐way ANOVA with Tukey's multiple comparison or Kruskall–Wallis test (^*^ *p* < 0.05; ^***^ *p* < 0.001; ^****^ *p* < 0.0001; ns, non‐significant). D) Proposed model of PIN3‐dependent regulation of nitrate uptake in soybean leaf pavement cells. In low‐nitrate conditions (left), *NPF2.13* (encoding a nitrate transporter localized to the plasma membrane) is activated by the light‐responsive transcription factor STF3, and PIN3 transporters promote auxin efflux at the plasma membrane. In high‐nitrate conditions (right), nitrate‐induced PIN3a ubiquitination and degradation occur to render PIN3a concentrated at tripartite cell–cell junctions, thereby down‐regulating auxin efflux at pavement‐cell junctions. Over‐accumulation of auxin in *pin3* mutants in high‐nitrate circumstances results in larger cell size and increased lobing. Meanwhile, auxin over‐accumulation results in activation of *ARFs*, particularly *ARF10*, to further stimulate NPF2.13‐mediated nitrate transport. ARF10 also enhances photosynthetic efficiency through STF3/4‐dependent light signaling, which in turn further amplifies *NPF2.13* expression. *ARF10–NPF2.13*, *STF3–ARF10*, and *STF3–NPF2.13* genetic modules contribute to balancing nitrate uptake, auxin efflux, and photosynthetic performance under high‐nitrate conditions.

To assess the tolerance of *pin3* mutants to low‐nitrate conditions, we conducted a comparative growth study of WT and *pin3ab* mutants in the field in Sanming, China, in 2024 without fertilizer application. *pin3ab* mutants had a statistically significant enhancement in photosynthetic efficiency and nitrogen content in both leaves and seeds (**Figure** **7**A,B). Although *pin3ab* had slightly decreased yield under nitrate starvation in the field, these mutants showed an increase of 2.3 percentage points in seed‐oil content, with no adverse effect on protein content (Figure 7B).

To assess the broader agronomic impact of nitrogen over‐accumulation and enhanced photosynthesis on soybean yield traits and seed composition, we conducted two‐year field trials with WT and *pin3ab* mutants in southern China at four sites spanning 8° of latitudinal difference: Putian (2023), Sanya (2024), Sanming (2024) and Fuzhou (2024), according to normal growth conditions with application of nitrogen fertilizer. Throughout these trials, *pin3ab* mutants consistently displayed higher SPAD values compared to WT (Figure 7C; Figure S13A, Supporting Information). Due to variation in photoperiod and growth across these field sites, plant height for *pin3ab* double mutants was higher than WT in Putian and Sanming, and *pin3ab* had more seeds compared to the WT in Putian. 100‐seed weights were statistically significantly lower in *pin3ab* from WT in Putian and Sanming (Figure S13B, Supporting Information). The comparable seed‐weight per plant between WT and *pin3ab* (in Putian and Sanming) can be attributed to smaller but more seeds produced by *pin3ab* mutants (Figure S13B, Supporting Information). Seed‐yield per square meter for *pin3ab* was statistically non‐significantly different from WT (Figure 7C).

Nitrogen and carbohydrate (from photosynthesis) are exported for long‐distance transport to seeds to support the production of seed proteins and oil.^[^ ^43^, ^44^ ^]^ Transgenic lines overexpressing the oleosin protein‐encoding gene (*GmOLEO1*) have a statistically significant average increase of 2.12 percentage points in seed‐oil content compared to WT seeds.^[^ ^45^ ^]^ Similarly, high‐oil‐type gene‐edited *GmSWEET10b* lines (*sw10b‐ho‐1* and *sw10b‐ho‐2*) showed statistically significant increases of 0.8–1.83 percentage points in oil content compared to WT seeds.^[^ ^46^ ^]^ Here, *pin3ab* double mutants had a statistically significant increase in seed‐oil content with 1.03–2.10 percentage points without impacting protein contents across four locations and two years (Figure 7C). Given that improving oil content by 2–3 percentage points in soybean is extremely challenging, these findings demonstrate a robust molecular‐breeding approach via disruption of *PIN3* function that leads to improved soybean seed oil without major growth or yield trade‐offs.

## 3 Discussion

Enhancing nitrogen‐use efficiency in soybean and other staple crops is a priority for achieving sustainable agriculture. Here, we identified a role for the soybean auxin‐efflux transporters PIN3a and PIN3b in the acquisition of nitrate. PIN3a is localized to cell junctions in soybean leaves, and high‐nitrate conditions promote its relocalization and accelerate its degradation. Deficiency of PIN3a and select homologs results in the over‐accumulation of auxin, which in turn activates auxin signaling, STF3/4 signaling, and nitrate‐transport pathways (Figure 7D). In the absence of nitrogen fertilizer, *pin3ab* mutants had more nitrogen in sink organs in the field. This suggests that soybean plants with *PIN3a/b* impairment, which display high nitrogen‐use efficiency, are potentially suitable for cultivation in nitrogen‐deficient soil. These findings underscore the significance of PIN3‐mediated auxin transport in facilitating nitrate uptake for optimal soybean growth and the complexity of transcription‐factor networks in plants.

Nitrate transport and signaling pathways are known to intersect with the auxin network,^[^ ^47^ ^]^ with Arabidopsis NPF6.3 transporting both nitrate and auxin.^[^ ^8^, ^48^, ^49^ ^]^ Under low‐nitrate concentrations, NPF6.3 contributes to the regulation of auxin biosynthesis through TRYPTOPHAN AMINOTRANSFERASE RELATED 2 and transport via LIKE AUX1 3.^[^ ^50^ ^]^ Nitrate impacts the activity and localization of PIN2 and PIN7 for auxin efflux.^[^ ^51^ ^]^ These studies highlight the hierarchical relationship between nitrate and auxin transport, with nitrate supply having major effects across numerous aspects of plant development. Auxin over‐accumulation in leaf pavement cells in *pin3* mutants stimulates light signaling through an ARF10–STF3/4 pathway and stimulates nitrate transport through ARF–NPF and STF–NPF genetic modules. We observed that high‐nitrate levels expedited the degradation of PIN3a via proteasome‐mediated ubiquitination. However, the specific interacting protein/s responsible for PIN3a degradation remain unknown. NPF6.3B interacts with the SPX‐domain‐containing protein SPX4 in a nitrate‐dependent manner, leading to the ubiquitination and degradation of SPX4 through the recruitment of NPF6.3B‐INTERACTING PROTEIN 1, an E3 ubiquitin ligase.^[^ ^52^ ^]^ We therefore hypothesized that high‐nitrate levels might facilitate PIN3a degradation by forming a complex with an E3 ubiquitin ligase. Nitrate exerts a negative effect on PIN3a abundance, while PIN3a, in turn, negatively regulates NPF‐dependent nitrate transport. However, it is crucial to have a “brake” mechanism in place to control this amplification process.

Excessive nitrogen‐fertilizer application to soybean diminishes nitrogen‐fixation capacity, leading to fewer nodules and suppressed organogenesis, reduced nitrogenase activity, and accelerated nodule senescence.^[^ ^53^, ^54^, ^55^ ^]^ This suggests that soybean uses nitrogen fixation and nitrate uptake as contrasting strategies to meet nitrogen requirements, with uptake favored when external supply is abundant. The impaired nitrogen‐fixation capacity observed in the *pin3ab* and *pin3abd* mutants may indicate a trade‐off mechanism to balance the heightened nitrate‐uptake capacity in *pin3* mutants, owing to the intensive carbon demands of symbiotic nitrogen fixation. These dual negative regulations offer potential for enhancing nitrogen‐use efficiency in soybean and potentially other legumes.

Auxin plays a crucial role in the establishment of leaf pavement‐cell polarity.^[^ ^27^, ^56^ ^]^ During polarity establishment, localized high levels of auxin activate the Rho family of ROP GTPases that promote cell interdigitation. Activated ROP GTPases enhance the polar distribution of PIN1 by inhibiting its endocytosis.^[^ ^32^ ^]^ Consequently, polarized PIN1 and auxin act synergistically to promote pavement‐cell polarity. We identified PIN3a as a negative regulator of nitrate‐mediated pavement‐cell expansion, and soybean *pin3ab* and *pin3abd* mutants over‐accumulate auxin and have ectopic auxin signaling. However, contrary to the expected negative role of PIN3a/b in cell expansion, these mutants have smaller pavement cells compared to WT. This discrepancy can be explained by two possibilities. First, the localization of PIN3a/b in pavement cells may contribute to the early stages of lobe‐polarity formation, which could impact cell expansion. Second, the auxin buildup in *pin3ab* and *pin3abd* mutants might be sufficient to suppress leaf cell growth. Further investigation is necessary to understand the coordinated regulation of PIN1 and PIN3 in lobing polarity and pavement‐cell expansion.

PINs consist of two transmembrane domains at both the N‐ and C‐termini, with a hydrophilic loop in the middle.^[^ ^57^, ^58^ ^]^ While AtPIN3/4/7 exhibit a more widespread distribution surrounding the pavement‐cell membrane in Arabidopsis, GmPIN3a was predominantly present at tripartite cell‐wall junctions. GmPIN3 shares functional conservation with AtPIN3, however, the specific cellular distribution of this transporter piqued our interest in investigating its association with cell junctions. One potential explanation for the differences in PIN3 localization between Arabidopsis and soybean is the role of adherens junctions — rich in sterols and sphingolipids. These lipid‐raft components play a crucial role in the association of PIN proteins with the membrane.^[^ ^59^, ^60^, ^61^ ^]^ Lipid components and proportions vary across plant taxa, leading to localization diversity amongst GmPIN3s.

Although *pin3ab* mutants exhibit significant increases in nitrogen accumulation and photosynthetic efficiency, the elevated assimilate production does not translate into a proportional enhancement in seed yield. This decoupling of assimilate availability and yield output suggests that carbon allocation in soybean is subject to additional regulatory mechanisms beyond source strength. One possible explanation is that sink capacity—defined by the developmental and metabolic potential of seeds—may become limiting under enhanced photosynthetic conditions. In *pin3ab* mutants, the redirection of carbon toward oil biosynthesis, possibly via STF3/4‐mediated light signaling, may prioritize storage compound quality over quantity, thereby maintaining protein levels while increasing oil content without expanding overall seed biomass. Soybean seeds are rich in storage compounds, with oil and protein comprising ≈60% of their total content. The enhancement of these components is a cornerstone of soybean breeding, aiming to develop varieties with increased oil or protein content.^[^ ^62^ ^]^ Soybean oil is stored primarily as fatty acids, triacylglycerols, and tocopherols.^[^ ^62^ ^]^ Typically, high oil and protein content are inversely related traits. High oil content is often associated with lower protein content and vice versa, making it challenging to develop soybean varieties that excel in both aspects simultaneously. Our findings indicate that *pin3ab* double mutants have increased seed‐oil levels without changes to protein contents, suggesting that PIN3a and PIN3b play a role in energy allocation toward oil biosynthesis. The auxin gradient, mediated by PIN3 and previously characterized in Arabidopsis,^[^ ^63^ ^]^ may regulate key factors involved in soybean oil and protein accumulation. The up‐regulation of STF3/4‐mediated light signaling in *pin3ab* mutants indicates an enhanced capacity for carbon assimilation through photosynthesis, potentially explaining the observed increases in oil without a compromise in protein content and yield. Further studies ought to clarify the mechanisms by which these genes modulate oil biosynthesis and energy allocation.

## 4 Experimental Section

### Plant Material and Growth Conditions

The wild‐type (WT) soybean used in this study was the elite cultivar Hua‐Chun 6 (HC‐6), which is a dominant variety grown in southern China. For experiments in controlled settings, seeds were germinated and cultivated in vermiculite at 25 °C (without rhizobia inoculation), a photoperiod of 14 h light and 10 h darkness, and a light intensity of 270 µmol m^−2^ s^−1^ of white light.

For indoor experiments, all high nitrate hydroponic assays were performed with 5.3 mm KNO_3_, a standard level routinely used for Arabidopsis.^[^ ^15^ ^]^ high‐nitrate treatment was performed as follows: seeds were germinated in vermiculite for 6 d, then transferred to either a low‐nitrogen buffer or a high‐nitrogen buffer for an additional 5 d. The low‐nitrogen buffer (without nitrate added) consisted of 5300 µm KCl, 1.2 mm CaCl_2_,  0.3 µm K_2_SO_4_, 500 µm MgSO_4_•7H_2_O, 25 µm MgCl_2_, 2.5 µm Na_2_B_4_O_7_•10H_2_O, 1.5 µm MnSO_4_•H_2_O, 1.5 µm ZnSO_4_•7H_2_O, 0.5 µM CuSO_4_•5H_2_O, 40 µM Fe‐Na‐EDTA, 11.8 µM Na_2_MoO_4_·2H_2_O，0.184 µM CoCl_2_·6H_2_O and 250 µM KH_2_PO_4_.^[^ ^29^ ^]^ The high‐nitrogen buffer contained 5.3 mm KNO_3_ instead of KCl, while the other components remained the same. These treatments were carried out at 26 °C under a photoperiod of 14:10 h light:dark.

For outdoor and field experiments, WT, *pin3ab*, *pin3abd^CR1,^* and *pin3abd^CR2^* mutant lines were planted with three replicates of each genotype in experimental fields in China from 2020–2024. Field experiments were carried out by growing plants for 100–120 days in different locations and time periods. Samples, including leaves, stems, seeds, and seed coats were collected from consistent areas of the plants.

- In Fujian province, Fuzhou outdoor experiments (26°08′ N 119°24′ E) were conducted by growing plants in cylindrical pots with a diameter of 45 cm and a depth of 28 cm for 90–110 d, planting ranging from 28–38 °C from April–November. Each pot contained one genotype plant, four plants were planted in each pot, and 8–10 g m^−2^ nitrogen was applied as fertilizer at the early flowering stage. For Fuzhou field experiments, plants were grown from July–October of 2024 with temperatures ranging from 20–38 °C. Each experiment contained three rows with a row length of 4 m and a spacing of 0.5 m between rows. There were 36 4‐m‐long rows per plot (≈4.8 m^2^). The growth density per plot was calculated as 25 plants per square meter, and 9.2 g m^−2^ nitrogen fertilizer was applied as a normal growth condition. Fertilizer was applied twice, in the early‐flowering and full‐flowering stage (Figure 7C; Figure S13, Supporting Information).
- In Fujian province, Sanming (26°28′ N 118°49′ E) field experiments, plants were grown from May–August of 2024 with temperatures ranging from 20–38 °C. Each experiment contained three rows with a row length of 4 m and a spacing of 0.5 m between rows. There were 36 4‐m‐long rows per plot (≈4.8 m^2^). The growth density per plot was calculated as 25 plants per square meter, and 9.2 g m^−2^ nitrogen fertilizer was applied as a normal growth condition. Fertilizer was applied twice, in the early flowering and full flowering stages. No fertilizer was used in Figure 7A.
- In Fujian province, Putian (25°32′ N 119°13′ E) field experiments, plants were grown from August–November in 2023 with temperatures ranging from 20–38 °C. Each experiment contained three rows with a row length of 13.5 m and a spacing of 0.5 m between rows. There were six 6‐m‐long rows per plot (≈12 m^2^). The growth density per plot was calculated as 30–40 plants per square meter, and 19.2 g m^−2^ nitrogen fertilizer was applied as a normal growth condition. Fertilizer was applied once during seed germination (Figure 7C; Figure S13, Supporting Information).
- In Hainan province, Sanya (18°32′ N 109°46′ E) field experiments, plants were grown from December 2023 to February 2024 with temperatures ranging from 20–33 °C. Each experiment contained 12 rows with a row length of 1.5 m and a spacing of 0.5 m between rows. The plots were designed as 12 1.5‐m long rows per plot (≈9 m^2^). The growth density per plot was calculated as 30–50 plants per square meter, and 13.49 g m^−2^ nitrogen fertilizer was applied as a normal growth condition. Fertilizer was applied once during seed germination (Figure 7C; Figure S13, Supporting Information).

*Arabidopsis thaliana* seeds were sterilized in a desiccator jar with a 100‐mL beaker containing 45 mL sodium hypochlorite (NaOCl) and 5 mL concentrated hydrochloric acid for 2 h, then sown on agar plates containing half‐strength Murashige & Skoog medium (½ MS) supplemented with 0.8% agar. Plates were kept at 22 °C under a photoperiod of 16 h light at 200 µmol m^−2^ s^−1^ and 8 h darkness. After 5 d of growth, seedlings of the wild type (Columbia‐0), *pin3‐4* mutant^[^ ^64^ ^]^ and *AtPIN3_pro_:GmPIN3–GFP* (in the *pin3‐4* background) were used for gravitropism analysis. Seedlings were vertically grown in darkness for 4 d following germination, and the angle of hypocotyl growth was measured by reorienting the hypocotyls horizontally for 24 h.

*Nicotiana benthamiana* seeds (tobacco) were grown in a growth chamber under a 14 h light/10 h dark photoperiod at 25 °C. 5–6‐week‐old plants were used for infiltration with *Agrobacteria*.

### Molecular Cloning

For CRISPR–Cas9‐based gene editing, sgRNAs were designed using CRISPR‐GE (http://skl.scau.edu.cn/home/). Vector construction was performed using pGES201 and pGES401 as previously described.^[^ ^65^, ^66^ ^]^ Transgenic plants containing *GmPIN3(a–d)_pro_:GUS* and *Super_pro_:STF3–GFP* constructs were generated previously.^[^ ^29^, ^67^ ^]^ To construct *Super_pro_:ARF10–GFP* vectors, the *ARF10* coding sequence was amplified from cDNA obtained from c.v. HC‐6 leaves. PCR products were recombined into the pSuper‐N‐GFP vector.^[^ ^67^ ^]^ All primer sequences and vectors used in this study are found in Table S5 (Supporting Information).

### Generation of Stable‐Transgenic Soybean Plants

Constructs described above were introduced into *Agrobacterium tumefaciens* EHA101 or GV3101. The soybean transformation protocol was followed as previously reported.^[^ ^65^ ^]^ Resulting transformants were screened by PCR using primers targeting the herbicide‐resistance *BAR* gene and the corresponding genes of interest. Primers used are found in Table S5 (Supporting Information).

### Chemical Treatments

1‐Naphthlcetic acid (NAA, Sigma, 317 918) was dissolved in 100 µL of 1 mol L^−1^ sodium hydroxide then fully dissolved in ultrapure H_2_O as a stock solution for pharmacological treatment. N‐1‐naphthylphthalamic acid (NPA, TCI, N0067), brefeldin A (BFA, sigma, 20350‐15‐6), FM4‐64 (Sigma–Aldrich, SCT127), cycloheximide (CHX, INALCO, 1758‐9310) and MG132 (Selleck, S2619) were dissolved in DMSO as a stock solution for pharmacological treatment. Each stock solution was further dissolved with ultrapure H_2_O and sprayed on leaves with 0.1% v/v Tween 20.

### Tissue Sectioning and Microscopy Observation

For histochemical analysis of GUS expression, leaves were collected and placed in staining solution comprising 50 mM sodium phosphate buffer (pH 7.0), 0.1% (v/v) Triton X‐100, 0.1 mm K_3_Fe(CN)_6_, 0.1 mm K_4_[Fe(CN)_6_]•3H_2_O, 1 mg mL^−1^ X‐Gluc and 1% (v/v) dimethylformamide. Leaves were incubated at 37 °C for 1–8 h then washed in 70% (v/v) ethanol. All samples in a single experiment were stained for a consistent duration. Images were captured using a Nikon ECLIPSE Ni‐U light microscope.

For pavement‐cell analysis, a previously described method^[^ ^27^ ^]^ was followed. True leaves from 10‐d‐old plants were cut into small pieces and treated with a 4:1 mixture of ethanol and acetic acid to remove chlorophyll. Leaves were then immersed in a solution containing 7% (w/v) NaOH in 60% (v/v) ethanol. Finally, leaves were briefly washed with 40% (v/v) ethanol and imaged using light microscopy (Nikon ECLIPSE Ni‐U). Pavement‐cell‐shape analysis was performed using Image J. Scanning‐electron‐microscopy observation was conducted using a TM‐3030 table‐top scanning electron microscope (Hitachi) at an accelerating voltage of 15 kV.

For fluorescence observation, images were captured using Leica SP8 confocal microscopes. Leaves from 11‐d‐old plants were cut into small pieces and directly used for imaging. The settings for excitation and detection were as follows: GFP: 488 nm excitation, 507 nm emission; calcofluor white: 405 nm excitation, 430–500 nm emission; Venus: 514 nm excitation, 524–555 nm emission. All images in a single experiment were taken using the same settings.

Sample staining and microscopy observation included the following methods:

1) Immunostaining with the anti‐IAA antibody was performed as described and adapted from the previous studies.^[^ ^68^, ^69^ ^]^ Anti‐IAA antibody (Agrisera, 1:300) was used as the primary antibody and Alexa Fluor 488 goat anti‐rabbit (Invitrogen, 1:300) was used as the secondary antibody. Immunostaining procedures were performed on slides.

2) Calcofluor white staining: Leaves were placed in 50% (v/v) glycerol containing 0.15% w/v calcofluor white for 20 min before sealing with 50% (w/v) glycerin and observed with confocal microscopy.

3) Brefeldin A treatment: leaves of 6‐days‐old *PIN3a_pro_:PIN3a–GFP* transgenic soybean plants were placed in low nitrogen or high nitrogen‐containing buffer for another 5 d. Leaves were cut into squares and immersed in 200 µm brefeldin A in darkness for 2 h and GFP signal was imaged by confocal microscopy. For FM4‐64 co‐staining, FM4‐64 dye was added to a final concentration of 2 µL mL^−1^ and leaves were stained for another 10 min.

4) TIRF imaging: leaves of 7‐days‐old *PIN3a_pro_:PIN3a–GFP* transgenic soybean plants were transferred to low‐nitrogen or high‐nitrogen‐containing buffer for another 5 d and imaged by Multi‐TIRF (wide field). Imaging mode in Multimodality Structured Illumination Microscopy (Mutli‐SIM) imaging software, select 488 nm laser, set laser to 80% (100 mW) and the imaging exposure time to 5 × 10 ms (imaging time per frame). The time‐series parameter was set to 1000 cyc (no time interval), the focus lock was set (PFS on) for image acquisition. After data acquisition, Deconv (parameter‐Auto) in reconstruction software was used for deconvolution processing.^[^ ^70^ ^]^

### IAA Quantification by UPLC

Auxin extraction was performed as described previously^[^ ^29^, ^71^ ^]^ with some modifications. The sample was ground into powder with liquid nitrogen. A mixture of 900 µL ethyl acetate and 100 µL internal standard was added to 20 mg of powder. After mixing, samples were ultrasonically extracted for 20 min at 4 °C, the supernatant was transferred to a new centrifuge tube and evaporated to dryness with a cold‐trap concentrator (Eppendorf, Germany). The dry residues were dissolved in 200 µL of 70% methanol. The resulting solution was then filtered using a 0.22 µm PVDF syringe filter before being subjected to UPLC‐XEVO TQS analysis. After injection into a UPLC system coupled with a triple‐quadrupole mass spectrometer XEVO TQ‐S (Waters, USA), samples were separated on an Acquity BEHC18 column (2.1 × 100 mm, 1.7 µm, Waters) at 40 °C with mobile phase A (5 mM ammonia acetate) and mobile phase B (methanol) running a gradient elution (0–2.5 min, 5% B–20% B, 3 min, 45% B, 4–5 min 100% B and returned to the initial maintained for 3 min) at a flow rate of 0.3 mL min^−1^. Auxin was detected with MRM scanning mode and quantified with a calibration curve after matrix effect correction with comparisons of ion peak intensities with those of corresponding internal standards. The raw mass data were analyzed using the MassLynx software (version 4.1, Waters). Quantification was carried out with targetlynx in MassLynx software, followed by Microsoft Excel.

### ^15N^ Abundance Measurement

Soybean seeds were germinated in vermiculite for 6 d before transfer to a nutrient solution containing 5.3 mm K^15^NO_3_ with a 10.14% atomic excess of ^15^N for an additional growth. During this period, leaves and roots were sampled and dried in a 65 °C oven for 24 h.

For root‐to‐shoot ^15^N uptake (short‐term), soybean seeds were germinated in vermiculite for 13 d. After removing the cotyledons for 1 d, the seedings were transferred to a nutrient solution containing 5.3 mm K^15^NO_3_ with a 99% atomic excess of ^15^N for an additional 7 h, and then leaves were harvested and dried in a 65 °C oven for ^15^N measurement.

For root‐to‐shoot ^15^N uptake (long‐term), seeds were germinated in vermiculite for 6 d before transfer to a nutrient solution containing 5.3 mm K^15^NO_3_ with a 10.14% atomic excess of ^15^N for an additional 24 h of growth. Afterward, seedlings were transferred to a low‐nitrogen‐containing buffer for another 10 d, and leaves and roots were harvested and dried in a 65 °C oven for ^15^N measurement.

The ratio of ^15^N to ^14^N was measured using an Elemental Analyzer and Isotope Ratio Mass Spectrometer. Specific test parameters: REDOX tube temperature: 960°; Separation column temperature: 65°; Carrier gas flow rate: 100 mL min^−1^; Purge gas flow rate: 200 mL min^−1^; Oxygenation time 2 s; Injection mass: 2 mg. By comparing the results with an international standard substance (Atm‐N_2_), the ^15^N transport rate of the sample was calculated.

### ^15^NO_3_^−^ Uptake Assay in XENOPUS Oocytes

For expression in *Xenopus laevis* oocytes, the full‐length cDNA of *AtNPF6.3* and *GmNPF2.13* were cloned into the vector pNB1.^[^ ^72^ ^]^ The mMESSAGE mMACHINE (ThermoFisher) was used for cRNA synthesis. Oocytes were injected with distilled water (50 nL as control) and the experimental cRNA (50 nL) whose concentration was adjusted to 1,000 ng µL^−1^. After incubation in ND96 medium (96 mm NaCl, 1 mm MgCl_2_, 1 mm CaCl_2_, 10 mm MES, adjusted to pH 7.5 with Tris‐base) for 2 d at 16 °C, oocytes were treated for 4 h in ND96 medium (pH 7.4) containing 10 or 0.1 mm ^15^N‐nitrate (atom % ^15^N abundance, 99.9%). Oocytes were then washed six times in ND96 medium. After complete drying at 65 °C, batches of 5–8 oocytes were then analyzed for total‐nitrogen content and atom % ^15^N abundance by continuous‐flow mass spectrometry (ANCA‐GSL MS; PDZ Europa). Nitrate uptake/oocyte (ng) = (measured abundance – natural abundance) × weight of the oocytes (mg)/number of oocytes × 10 000.

### Nitrogen Quantification

Samples were taken during the seed‐filling stage. 200 mg samples were place in a 300 mL deboiling tube. 5 mL sulfuric acid was added to the moistened samples with rotation at 2 g. A small funnel with a curved neck was placed at the mouth of the deboiling tube and deboiled at 250 °C in a furnace. When a large amount of white smoke was generated, the temperature was increased to 400 °C. After the sample turned brown, it was cooled down. The sample was distilled in the SKD‐1800 Automatic Kjeldahl Nitrogen Analyzer to absorb the internal ammonia into the boric acid solution. Total‐nitrogen content was determined according to the methyl red‐bromocresol green indicator. Total nitrogen (N) content is expressed in g/kg and calculated as follows: Total nitrogen (N) = (concentration of the standard sulfuric acid solution) × (the volume of the acid standard solution used for titrating the sample –the volume of standard acid solution used for blank titration) × 14.01 / sample weight. For high‐nitrate/rhizobia‐co‐supplied samples niteogen concentration:WT, *pin3ab*, and *pin3abd^CR2^* seedlings were germinated in vermiculite for 5 d, watered with low‐nitrogen buffer or a high‐nitrogen buffer for 3 d, treated with rhizobia for 4 d, and then transferred to low‐nitrogen buffer or a high‐nitrogen buffer for an additional 11 d. Leaves were harvested and oven‐dried at 65 °C overnight. A 50 mg sample was weighed, and the nitrogen content was determined using a carbon and nitrogen elemental analyzer(VARIO MAX, Germany).

### Photosynthesis Measurements

Net photosynthetic rate and stomatal conductance were measured in leaves of field‐grown soybean with a LICOR‐6800 Portable Photosynthesis System instrument at the flowering stage. The measurements were performed in CO_2_ gas between 8:30am and 11:30am in field experiments in summer and autumn.

### Nitrogenase Assays

To quantify nitrogenase activity, The authors used acetylene reduction activity (ARA).^[^ ^73^ ^]^ Fresh roots from 10 plants were harvested and individually placed into 50 mL glass vials that were sealed with rubber septa (1 root per glass vial). Subsequently, 5 mL of acetylene gas was injected into each vial and the vials were incubated at 28 °C for 2 h. To terminate the reaction, 5 mL of 1 m NaOH was added to each vial. To quantify ethylene production, a gas sample of 300 µL was extracted from each vial and analyzed using a gas chromatograph (GC‐2014). Column temperature is 100 °C; Inlet temperature is 150 °C; Detector temperature is 250 °C. Total nitrogenase activity was then calculated as ARA units represented by the number of nanomoles of ethylene produced per h per plant.

### Measurement of IAA Net Flux and Visualization of NO_3_^−^ Concentration

Net IAA flux and concentration gradients of NO_3_^−^ were determined using Non‐invasive Micro‐test Technology (NMT) (NMT Physiolyzer, Younger USA). The principles and methods of this NMT technique were described previously.^[^ ^74^ ^]^ Soybean plants were grown and treated in a growth chamber. IAA flux measurements were conducted using true leaves harvested from 7‐days‐old seedlings. The NMT measurement procedures were as follows: leaves were individually incubated in separate measuring solutions. For IAA measurement, 0.1 mm KNO_3_ at pH 6.0 was used, while for NO_3_^−^ concentration visualization, 1 mm KNO_3_ at pH 6.0 was used. The incubation period lasted 40 min. The IAA microsensor (XY‐CGQ‐503) and NO_3_^−^ microsensor (XY‐STZ‐NO_3_‐C) were calibrated using a range of standards that covered the measurement spectrum. The epidermal cells were found under the microscope, and the IAA and NO_3_^−^ concentrations microsensor was placed 5 µm above the epidermal cell layer to begin the detection. 5 min of data were recorded at each cell and 8 replicates were detected in each group. Net IAA fluxes and NO_3_^−^ concentrations were recorded and exported using imFluxes v3.0 software (Xuyue Company, China), and the necessary consumables were sourced from Xuyue Company (China).

For IAA detection,^[^ ^74^ ^]^ the epidermal cells were found under the microscope, and the IAA concentrations microsensor was placed 5 µm above the epidermal cell layer to begin the detection. 5 min of data were recorded at each cell and 8 replicates were detected in each group. For NO_3_^−^detection,^[^ ^75^ ^]^ the NO_3_^−^ flux microsensor was positioned extremely close to, but not touching, the surface of the epidermal tissue, which was marked as 0 µm, and the NO_3_^−^ concentration was recorded. Subsequently, the NO_3_^−^ sensor was moved away from the epidermal tissue at distances of 20, 40, 60, 80, 100, 120, 140, and 160 µm from the epidermis, and the NO_3_^−^ concentrations were recorded at each distance. Each group was tested with 9 biological replicates, and a spatial concentration map of NO_3_^−^ on the epidermal surface was generated. Net IAA fluxes and NO_3_^−^ concentrations were recorded and exported using imFluxes v3.0 software (Xuyue Company, China), and the necessary consumables were sourced from Xuyue Company (China).

### Transcriptomic Analysis

Soybean seeds were germinated in vermiculite for 6 d, then transferred to low‐ or high‐nitrogen conditions for an additional 5 d of growth in a growth chamber. Leaf samples were collected from a consistent area of true leaves, with each sample collected in triplicate. Samples were then ground into a fine powder using liquid nitrogen. Total RNA was extracted from the ground samples using the RNAprep Pure Plant Kit (Tiangen). mRNA sequencing was performed using the Illumina NovaSeq 6000 platform (Novogene, China).

To quantify expression levels, the Trinity reference was annotated, and paired‐end reads were mapped to the assembled transcripts and assigned to genes using RSEM v1.2.20. Differential‐expression analysis was conducted using the R package EdgeR v3.32.0. Expression levels were measured as counts per million (CPM). Genes with a fold change (LogFC) greater than or equal to 1 and a false‐discovery rate (FDR) below 0.05 were classified as differentially expressed genes (DEGs).

DEGs were mapped to gene‐ontology (GO) terms available in the GO database (http://www.geneontology.org/). Gene numbers associated with each GO term were calculated. Pathway analysis DEGs was performed using the Kyoto Encyclopedia of Genes and Genomes (KEGG) database (https://ngdc.cncb.ac.cn/gsub/). Statistical enrichment of DEGs for GO terms was conducted using agriGO v2.0, employing a hypergeometric test. Statistically significantly enriched GO terms and KEGG pathways (corrected *p*‐value < 0.05) were identified based on these analyses.^[^ ^76^, ^77^ ^]^ Raw transcriptome data have been uploaded in the NGDC database (Number: PRJCA036084).

### Phylogenetic Analyses

The sequence of PIN proteins from Arabidopsis, Medicago, soybean was obtained from Phytozome (https://phytozome‐next.jgi.doe.gov). Amino‐acid sequences were aligned using Clustal X.^[^ ^78^ ^]^ A phylogenetic tree was constructed using MEGA X^[^ ^79^ ^]^ with neighbor‐joining (NJ) criteria and verified using the maximum likelihood (ML). A bootstrap test (1000 replicates) was conducted based on multiple alignments of PIN sequences.

### Dual‐Luciferase Reporter Assays

Dual‐luciferase reporter assays were performed following a previously described protocol.^[^ ^80^ ^]^ 3.2‐kb *STF3* and *NPF2.13* promoters were cloned into pGreenII 1300‐LUC^[^ ^67^ ^]^ with LUC and REN reporters. Reporter constructs were transformed into *A. tumefaciens* GV3101. Strains containing the reporter constructs and the transcription‐factor effector strains (OD_600_ = 0.8) were mixed in a 1:1 ratio and co‐infiltrated into *N. benthamiana* leaves. Infiltrated leaves were cultivated in a growth chamber under 14‐h light at 200 µmol m^−2^ s^−1^ white light/10‐h darkness at 26 °C. Two days after infiltration, LUC and REN activities were assayed using the Dual‐LUC Reporter Assay System (Cat. no. 11402ES60, YEASEN, China). The relative luciferase activity was calculated as the ratio of firefly luciferase to Renilla luciferase (LUC/REN).

*ChIP–qPCR*: The full‐length *ARF10* coding sequence was cloned into *Super_pro_‐N‐GFP* ^[^ ^67^ ^]^ and transformed into *A. tumefaciens* GV3101 for transient expression in soybean leaves. Based on the transient transformation method for soybean leaves,^[^ ^81^ ^]^ soybean seedlings were grown under a 14‐h light/10‐h dark photoperiod in a growth room at 26 °C for 10 days then the epidermis of the leaf was wiped with a brush. The transformed *Agrobacterium* cells were adjusted to OD_600_ = 0.6 with infiltration buffer [10 mm MES, pH 5.6, 200 µm acetosyringone (Sigma)] and pressure‐infiltrated into the leaves using a vacuum pump until the leaves were completely wet. The seedlings were left in the continuous darkness to recover for another 24 h and then grown under normal conditions.

For ChIP assays, 14‐days‐old leaves from stable‐transgenic *Super_pro_:STF3–GFP* plants were collected. ≈2 g of leaf tissue was used following a previously described protocol.^[^ ^82^ ^]^ In short, leaves were cross‐linked under vacuum in a crosslinking buffer containing 1% (v/v) formaldehyde for 20 min, and 100 mm glycine was added and incubated for another 10 min. After washing four times with distilled water, samples were used for chromatin isolation, sonication, and immunoprecipitation with anti‐GFP agarose beads (Cat. no. AE074, ABclonal Technology) at 4 °C overnight with gentle rotation. Precipitated chromatin was eluted, de‐crosslinked by 5 mm NaCl, and isolated with an equal volume of phenol:chloroform:isoamyl alcohol (25:24:1) then analyzed by quantitative PCR (qPCR). Specific ChIP–qPCR primers were designed to amplify promoter sequences of *STF3* and *NPF2.13* (Table S5, Supporting Information). Real‐time quantitative polymerase chain reaction (real‐time qPCR) was performed in a total volume of 20 µL of reaction buffer (2x SYBR mix, 10 µL; primer‐F (10 µmol), 0.4 µL; primer‐R (10 µmol), 0.4 µL; H_2_O, 7.2 µL; template DNA, 2 µL) by a Bio‐Rad CFX96 real‐time system (Bio‐Rad). Unprecipitated ultrasound chromatin DNA was used as a negative input control and ChIP results were presented as a percentage of input DNA.

### EMSA

The DNA‐binding domain sequence of *ARF10* was cloned into the pCold‐His vector and introduced into *Escherichia coli Rosetta2 (DE3)* pLysS. Protein induction was achieved by adding IPTG (0.5 mm) at 16 °C for 24 h in lysogeny broth. Subsequently, 6xHis–ARF protein was purified using the His‐tag Protein Purification system (Cat. no. 70 601, BEAVER, China). Similarly, the *STF3* coding sequence was cloned into the pET28a‐His vector and introduced into *E. coli* BL21. Protein induction was carried out by adding IPTG (0.25 mm) at 37 °C for 4 h in lysogeny broth.

6xHis–STF3 was purified using the His‐tag Protein Purification system as for 6xHis–ARF10. For the binding assay, labeled probes (0.2 µm) were incubated with recombinant candidate proteins in binding buffer, following the instructions of the LightShift Chemiluminescent EMSA Kit (Cat. no. 20 148, ThermoFisher Scientific, USA) at 25 °C for 30 min. The reaction mixture was loaded into a 4% non‐denaturing polyacrylamide gel in 0.5× TBE buffer and electrophoresed at 120 V for 60 min. Subsequently, the gel contents were transferred to a positively charged nylon membrane at 380 mA for 50 min and subjected to UV cross‐linking for 1 min. Products were detected using chemiluminescence with the LightShift Chemiluminescent EMSA Kit. The competitors were the double‐stranded oligonucleotide without any modification. Mutations of the double‐stranded oligonucleotide were that all motif bases changed to A.

### Western Blotting

*PIN3a_pro_:PIN3a–GFP* transgenic seedlings were germinated in vermiculite for 6 d before transferring to low‐ or high‐nitrogen containing solution for an additional 5 d. For the PIN3a protein degradation assay, leaves were then ground with liquid nitrogen and the resulting powder mixed with an equal volume of 2x loading buffer (0.4 m Tris‐HCl (pH 6.8), 4% (w/v) SDS, 0.2% (w/v) BPB, 20% (v/v) glycerin, and 2% (v/v) 2‐ME). For ubiquitination assays, leaves were ground with liquid nitrogen and immunoblotted with GFP magnetic beads (Cat. # L1016, Biolinkedin, China). The samples were heated at 65 °C for 10 min, then centrifuged at 14 000 *g* at 4 °C for 30 min. The proteins were mixed with loading buffer at a 1:1 ratio for gel loading.

Western blotting was performed following the manufacturer's instructions (Bio‐Rad). Proteins were separated by electrophoresis using 10% (w/v) SDS–PAGE gels and transferred to polyvinylidene fluoride (PVDF) membranes. Primary antibodies, including anti‐GFP (dilution 1:4000, Cat. # SAB2702211, Sigma–Aldrich, USA), anti‐Actin (dilution 1:4000, Cat. # A1978, Sigma–Aldrich, USA), anti‐Ub (dilution 1:1000, Cat. # 3936s, Cell Signaling Technology, USA) and anti‐K63 (dilution 1:1000, Cat. # 5621s, Cell Signaling Technology, USA), were used. For secondary‐antibody detection, either goat anti‐mouse IgG (H+L)–HRP (dilution 1:4000, Cat. no. A4416, Sigma–Aldrich, USA) or goat anti‐rabbit IgG (H+L)–HRP (dilution 1:1000, Cat. # 7074, Cell Signaling Technology, USA) was used. Signals were detected using an ECL Kit (Cat. # 20 148, Thermo Fisher Scientific, USA).

### Seed Oil and Protein Quantification

Seeds were randomly selected for the quantification of oil and protein. Seed oil and protein levels were determined by MATRIX‐I Fourier‐transform near‐infrared reflectance spectroscope (Bruker Optics). First, the MATRIX‐I was preheated for at least 30 min. Plump and uniform dry seeds from each plant were distributed in the sample container with no spaces remaining. The samples were scanned to obtain spectral data for determining the content of protein and oil. By using the dry‐basis model,^[^ ^83^ ^]^ protein and oil content were determined according to the Quant 2 method with OPUS v.5.5 software (en/products‐and‐solutions/ infrared‐and‐raman/opus‐ spectroscopy‐software/ downloads.html) (Bruker MPA).

### Yield Quantification

At full maturity, ≈15 randomly selected individual plants per line per plot were harvested. Four agronomic traits were scored: plant height, 100‐seed weight, seed‐weight per plant, and seed‐number per plant.

### Statistical Analysis

Camera and confocal‐microscopy images were prepared and quantified using ImageJ (http://imagej.nih.gov/ij/). Statistical data were analyzed in GraphPad Prism version 7. *p*‐values were calculated by one‐way or two‐way ANOVA with a false‐discovery rate of comparisons among multiple samples. The sample sizes, error bars, and statistically significant differences are stated in the respective figure legends.

## Conflict of Interest

Patents entitled “Auxin transporters GmPIN3 and its applications in soybean (ZL202410054994.9)” and “Application of auxin transporter *GmPIN3* gene family in the improvement of soybean breeding (202 410 455 656.6)” have been authorized in China. X.C., H.X., S.H., L.H., and J.C. are listed as inventors. The authors declare no other competing interests.

## Author contributions

X.C. and H.X. designed research. H.X., S.H., T.W., Q.H., K.W., T.T. and M.W. performed the research. Z.G. contributed RNA‐seq data analysis. H.X., J.W., X.S., Y.Z. and G.L. performed field experiments. L.H. and J.C. generated the transgenic soybean plants. Y.L. conducted NMT experiments. Z.C. contributed technical help and discussion. X.C. and H.X. wrote the paper.

## Supporting information



Supporting Information

Supporting Information

Supporting Information

Supporting Information

Supporting Information

Supporting Information

## Data Availability

The data that support the findings of this study are available from the corresponding author upon reasonable request.

## References

[1] W. Adalibieke , X. Cui , H. Cai , L. You , F. Zhou , Sci. Data 2023, 10, 617. 37696817 10.1038/s41597-023-02526-z PMC10495426

[2] R. Brackin , T. Näsholm , N. Robinson , S. Guillou , K. Vinall , P. Lakshmanan , S. Schmidt , E. Inselsbacher , Sci. Rep. 2015, 5, 15727. 26496834 10.1038/srep15727 PMC4620560

[3] J. N. Galloway , A. R. Townsend , J. W. Erisman , M. Bekunda , Z. Cai , J. R. Freney , L. A. Martinelli , S. P. Seitzinger , M. A. Sutton , Science 2008, 320, 889. 18487183 10.1126/science.1136674

[4] X. Liu , B. Hu , C. Chu , J. Genet. and Genom. 2022, 49, 394. 10.1016/j.jgg.2021.12.006 34973427

[5] Y. Y. Wang , Y. H. Cheng , K. E. Chen , Y. F. Tsay , Annu. Rev. Plant Biol. 2018, 69, 85. 29570365 10.1146/annurev-arplant-042817-040056

[6] Y. Y. Wang , P. K. Hsu , Y. F. Tsay , Trends Plant Sci. 2012, 17, 458. 22658680 10.1016/j.tplants.2012.04.006

[7] J. Sun , J. R. Bankston , J. Payandeh , T. R. Hinds , W. N. Zagotta , N. Zheng , Nature 2014, 507, 73. 24572362 10.1038/nature13074 PMC3968801

[8] G. Krouk , B. Lacombe , A. Bielach , F. Perrine‐Walker , K. Malinska , E. Mounier , K. Hoyerova , P. Tillard , S. Leon , K. Ljung , E. Zazimalova , E. Benkova , P. Nacry , A. Gojon , Dev. Cell 2010, 18, 927. 20627075 10.1016/j.devcel.2010.05.008

[9] A. Vega , I. Fredes , J. O'Brien , Z. Shen , K. Ötvös , R. Abualia , E. Benkova , S. P. Briggs , R. A. Gutiérrez , EMBO Rep. 2021, 22, 51813. 10.15252/embr.202051813 PMC8447600 34357701

[10] Y. Wang , Z. Yuan , J. Wang , H. Xiao , L. Wan , L. Li , Y. Guo , Z. Gong , J. Friml , J. Zhang , Proc. Natl. Acad. Sci. USA 2023, 120, 2221313120. 10.1073/pnas.2221313120 PMC10288568 37307446

[11] R. Abualia , K. Ötvös , O. Novák , E. Bouguyon , K. Domanegg , A. Krapp , P. Nacry , A. Gojon , B. Lacombe , E. Benková , Proc. Natl. Acad. Sci. USA 2022, 119, 2122460119. 10.1073/pnas.2122460119 PMC9351359 35878040

[12] H. Ishida , M. Izumi , S. Wada , A. Makino , Bioch. Biophys. Acta 2014, 1837, 512. 10.1016/j.bbabio.2013.11.009 24269172

[13] J. R. Evans , V. C. Clarke , J. Exp. Bot. 2019, 70, 7.30357381

[14] N. Gonzalez , H. Vanhaeren , D. Inze , Trends Plant Sci. 2012, 17, 332. 22401845 10.1016/j.tplants.2012.02.003

[15] S. Moreno , J. Canales , L. Hong , D. Robinson , A. H. K. Roeder , R. A. Gutiérrez , Curr. Biol. 2020, 30, 1988. 32302589 10.1016/j.cub.2020.03.036

[16] H. Sakakibara , J. Plant Res. 2003, 116, 253. 12836045 10.1007/s10265-003-0097-3

[17] J. S. Rubio‐Asensio , A. J. Bloom , J. Exp. Bot. 2017, 68, 2611. 28011716 10.1093/jxb/erw465

[18] S. Chaillou , J. W. Rideout , C. D. Raper Jr, J. F. Morot‐Gaudry , Physiol. Plant. 1994, 90, 259. 11537975

[19] P. Xu , E. Wang , Curr. Biol. 2023, 33, 543. 37279688 10.1016/j.cub.2023.04.053

[20] A. K. Spartz , H. Ren , M. Y. Park , K. N. Grandt , S. H. Lee , A. S. Murphy , M. R. Sussman , P. J. Overvoorde , W. M. Gray , Plant Cell 2014, 26, 2129. 24858935 10.1105/tpc.114.126037 PMC4079373

[21] W. Lin , X. Zhou , W. Tang , K. Takahashi , X. Pan , J. Dai , H. Ren , X. Zhu , S. Pan , H. Zheng , W. M. Gray , T. Xu , T. Kinoshita , Z. Yang , Nature 2021, 599, 278. 34707287 10.1038/s41586-021-03976-4 PMC8549421

[22] L. Hoermayer , J. C. Montesinos , P. Marhava , E. Benková , S. Yoshida , J. Friml , Proc. Natl. Acad. Sci. USA 2020, 117, 15322. 32541049 10.1073/pnas.2003346117 PMC7334516

[23] S. Vanneste , Y. Pei , J. Friml , Nat. Rev. Mol. Cell Biol. 2025, 26, 648. 40389696 10.1038/s41580-025-00851-2

[24] C. Luschnig , J. Friml , Nat. Commun. 2024, 15, 9904. 39548100 10.1038/s41467-024-54240-y PMC11567971

[25] A. W. Woodward , B. Bartel , Ann. Bot. 2005, 95, 707. 15749753 10.1093/aob/mci083 PMC4246732

[26] S. Zhang , L. Zhu , C. Shen , Z. Ji , H. Zhang , T. Zhang , Y. Li , J. Yu , N. Yang , Y. He , Y. Tian , K. Wu , J. Wu , N. P. Harberd , Y. Zhao , X. Fu , S. Wang , S. Li , Plant Cell 2021, 33, 566. 33955496 10.1093/plcell/koaa037 PMC8136903

[27] T. Xu , N. Dai , J. Chen , S. Nagawa , M. Cao , H. Li , Z. Zhou , X. Chen , R. De Rycke , H. Rakusová , W. Wang , A. M. Jones , J. Friml , S. E. Patterson , A. B. Bleecker , Z. Yang , Science 2014, 343, 1025. 24578577 10.1126/science.1245125 PMC4166562

[28] P. Grones , M. Majda , S. M. Doyle , D. Van Damme , S. Robert , Proc. Natl. Acad. Sci. USA 2020, 117, 16027. 32571946 10.1073/pnas.2007400117 PMC7355022

[29] Z. Gao , Z. Chen , Y. Cui , M. Ke , H. Xu , Q. Xu , J. Chen , Y. Li , L. Huang , H. Zhao , D. Huang , S. Mai , T. Xu , X. Liu , S. Li , Y. Guan , W. Yang , J. Friml , J. Petrášek , J. Zhang , X. Chen , Plant Cell 2021, 33, 2981. 34240197 10.1093/plcell/koab183 PMC8462816

[30] Y. Wang , C. Chai , B. Valliyodan , C. Maupin , B. Annen , H. T. Nguyen , BMC Genomics 2015, 16, 951. 26572792 10.1186/s12864-015-2149-1 PMC4647520

[31] I. Blilou , J. Xu , M. Wildwater , V. Willemsen , I. Paponov , J. Friml , R. Heidstra , M. Aida , K. Palme , B. Scheres , Nature 2005, 433, 39. 15635403 10.1038/nature03184

[32] S. Nagawa , T. Xu , D. Lin , P. Dhonukshe , X. Zhang , J. Friml , B. Scheres , Y. Fu , Z. Yang , PLoS Biol. 2012, 10, 1001299. 10.1371/journal.pbio.1001299 PMC3317906 22509133

[33] J. Kleine‐Vehn , Z. Ding , A. R. Jones , M. Tasaka , M. T. Morita , J. Friml , Proc. Natl. Acad. Sci. USA 2010, 107, 22344. 21135243 10.1073/pnas.1013145107 PMC3009804

[34] P. Žádníková , J. Petrášek , P. Marhavý , V. Raz , F. Vandenbussche , Z. Ding , K. Schwarzerová , M. T. Morita , M. Tasaka , J. Hejátko , D. Van Der Straeten , J. Friml , E. Benková , Development 2010, 137, 607. 20110326 10.1242/dev.041277

[35] A. S. Murphy , A. Bandyopadhyay , S. E. Holstein , W. A. Peer , Annu. Rev. Plant Biol. 2005, 56, 221. 15862095 10.1146/annurev.arplant.56.032604.144150

[36] J. Leitner , J. Petrášek , K. Tomanov , K. Retzer , M. Parezová , B. Korbei , A. Bachmair , E. Zažímalová , C. Luschnig , Proc. Natl. Acad. Sci. USA 2012, 109, 8322. 22556266 10.1073/pnas.1200824109 PMC3361439

[37] L. Abas , R. Benjamins , N. Malenica , T. Paciorek , J. Wisniewska , J. C. Moulinier‐Anzola , T. Sieberer , J. Friml , C. Luschnig , Nat. Cell Biol. 2006, 8, 249. 16489343 10.1038/ncb1369

[38] O. K. Teh , I. Moore , Nature 2007, 448, 493. 17653191 10.1038/nature06023

[39] C. S. Westfall , C. Zubieta , J. Herrmann , U. Kapp , M. H. Nanao , J. M. Jez , Science 2012, 336, 1708. 22628555 10.1126/science.1221863

[40] D. R. Boer , A. Freire‐Rios , W. A. M. vanden Berg , T. Saaki , I. W. Manfield , S. Kepinski , I. López‐Vidrieo , J. M. Franco‐Zorrilla , S. C. de Vries , R. Solano , D. Weijers , M. Coll , Cell 2014, 156, 577. 24485461 10.1016/j.cell.2013.12.027

[41] Y. Xiao , L. Chu , Y. Zhang , Y. Bian , J. Xiao , J. Xiao , D. Xu , Front. Plant Sci. 2021, 12, 800989. 35111179 10.3389/fpls.2021.800989 PMC8801436

[42] X. Chen , Q. Yao , X. Gao , C. Jiang , N. P. Harberd , X. Fu , Curr. Biol. 2016, 26, 640. 26877080 10.1016/j.cub.2015.12.066

[43] L. Miao , S. Yang , K. Zhang , J. He , C. Wu , Y. Ren , J. Gai , Y. Li , New Phytologist 2020, 225, 1651. 31596499 10.1111/nph.16250 PMC7496907

[44] S. Wang , S. Liu , J. Wang , K. Yokosho , B. Zhou , Y.‐C. Yu , Z. Liu , W. B. Frommer , J. F. Ma , L.‐Q. Chen , Y. Guan , H. Shou , Z. Tian , Natl. Sci. Rev. 2020, 7, 1776. 34691511 10.1093/nsr/nwaa110 PMC8290959

[45] D. Zhang , H. Zhang , Z. Hu , S. Chu , K. Yu , L. Lv , Y. Yang , X. Zhang , X. Chen , G. Kan , Y. Tang , Y.‐Q. C. An , D. Yu , PLoS Genet. 2019, 15, 1008267. 10.1371/journal.pgen.1008267 PMC6645561 31291251

[46] J. Wang , L. Zhang , S. Wang , X. Wang , S. Li , P. Gong , M. Bai , A. Paul , N. Tvedt , H. Ren , M. Yang , Z. Zhang , S. Zhou , J. Sun , X. Wu , H. Kuang , Z. Du , Y. Dong , X. Shi , M. Li , D. Shukla , L. Yan , Y. Guan , Adv. Sci. 2025, 12, 2500290. 10.1002/advs.202500290 PMC12199397 40365797

[47] R. Abualia , S. Riegler , E. Benkova , Cells 2023, 12, 1613. 37371083 10.3390/cells12121613 PMC10297484

[48] E. Mounier , M. Pervent , K. Ljung , A. Gojon , P. Nacry , Plant Cell Environ. 2014, 37, 162. 23731054 10.1111/pce.12143

[49] X. Zhang , Y. Cui , M. Yu , B. Su , W. Gong , F. Baluska , G. Komis , J. Samaj , X. Shan , J. Lin , Plant Physiol. 2019, 181, 480. 31431511 10.1104/pp.19.00346 PMC6776865

[50] A. Maghiaoui , E. Bouguyon , C. Cuesta , F. Perrine‐Walker , C. Alcon , G. Krouk , E. Benková , P. Nacry , A. Gojon , L. Bach , J. Exp. Bot. 2020, 71, 4480. 32428238 10.1093/jxb/eraa242

[51] K. Ötvös , M. Marconi , A. Vega , J. O'Brien , A. Johnson , R. Abualia , L. Antonielli , J. C. Montesinos , Y. Zhang , S. Tan , C. Cuesta , C. Artner , E. Bouguyon , A. Gojon , J. Friml , R. A. Gutiérrez , K. Wabnik , E. Benková , EMBO J. 2021, 40, 106862. 10.15252/embj.2020106862 PMC7849315 33399250

[52] B. Hu , Z. Jiang , W. Wang , Y. Qiu , Z. Zhang , Y. Liu , A. Li , X. Gao , L. Liu , Y. Qian , X. Huang , F. Yu , S. Kang , Y. Wang , J. Xie , S. Cao , L. Zhang , Y. Wang , Q. Xie , S. Kopriva , C. Chu , Nat. Plants 2019, 5, 401. 30911122 10.1038/s41477-019-0384-1

[53] H. Nishida , T. Suzaki , Curr. Opin. Plant Biol. 2018, 44, 129. 29684704 10.1016/j.pbi.2018.04.006

[54] X. Wang , Z. Qiu , W. Zhu , N. Wang , M. Bai , H. Kuang , C. Cai , X. Zhong , F. Kong , P. Lü , Y. Guan , Nat. Commun. 2023, 14, 4711. 37543605 10.1038/s41467-023-40392-w PMC10404276

[55] J.‐S. Lin , X. Li , Z. Luo , K. S. Mysore , J. Wen , F. Xie , Nat. Plants 2018, 4, 942. 30297831 10.1038/s41477-018-0261-3

[56] J. Chen , F. Wang , S. Zheng , T. Xu , Z. Yang , J. Exp. Bot. 2015, 66, 4957. 26047974 10.1093/jxb/erv266 PMC4598803

[57] Y. Zhang , C. Hartinger , X. Wang , J. Friml , New Phytologist 2020, 227, 1406. 32350870 10.1111/nph.16629 PMC7496279

[58] T. Bennett , Trends Plant Sci. 2015, 20, 498. 26051227 10.1016/j.tplants.2015.05.005

[59] K. Shigetomi , Y. Ono , T. Inai , J. Ikenouchi , J. Cell Biol. 2018, 217, 2373. 29720382 10.1083/jcb.201711042 PMC6028530

[60] F. Roudier , L. Gissot , F. Beaudoin , R. Haslam , L. Michaelson , J. Marion , D. Molino , A. Lima , L. Bach , H. Morin , F. Tellier , J.‐C. Palauqui , Y. Bellec , C. Renne , M. Miquel , M. DaCosta , J. Vignard , C. Rochat , J. E. Markham , P. Moreau , J. Napier , J.‐D. Faure , Plant Cell 2010, 22, 364. 20145257 10.1105/tpc.109.071209 PMC2845409

[61] J. Pan , S. Fujioka , J. Peng , J. Chen , G. Li , R. Chen , Plant Cell 2009, 21, 568. 19218398 10.1105/tpc.108.061465 PMC2660622

[62] Z. Duan , Q. Li , H. Wang , X. He , M. Zhang , Front. Plant Sci. 2023, 14, 1160418. 36959925 10.3389/fpls.2023.1160418 PMC10028097

[63] H. Liu , Q. Luo , C. Tan , J. Song , T. Zhang , S. Men , Plant J. 2023, 113, 1259. 36648165 10.1111/tpj.16109

[64] H. Rakusová , J. Gallego‐Bartolomé , M. Vanstraelen , H. S. Robert , D. Alabadí , M. A. Blázquez , E. Benková , J. Friml , Plant J. 2011, 67, 817. 21569134 10.1111/j.1365-313X.2011.04636.x

[65] M. Bai , J. Yuan , H. Kuang , P. Gong , S. Li , Z. Zhang , B. Liu , J. Sun , M. Yang , L. Yang , D. Wang , S. Song , Y. Guan , Plant Biotech. J. 2020, 18, 721. 10.1111/pbi.13239 PMC7004907 31452351

[66] W. Lin , H. Kuang , M. Bai , X. Jiang , P. Zhou , Y. Li , B. Chen , H. Li , Y. Guan , Crop J. 2023, 11, 825.

[67] J. Chen , H. Xu , Q. Liu , M. Ke , Z. Zhang , X. Wang , Z. Gao , R. Wu , Q. Yuan , C. Qian , L. Huang , J. Chen , Q. Han , Y. Guan , X. Yu , X. Huang , X. Chen , New Phytologist 2024, 241, 209. 37881032 10.1111/nph.19353

[68] C. Bianco , B. Senatore , S. Arbucci , G. Pieraccini , R. Defez , Appl. Environ. Microbiol. 2014, 80, 4286. 24814784 10.1128/AEM.00597-14 PMC4068685

[69] M. Sauer , T. Paciorek , E. Benkova , J. Friml , Nat. Protoc. 2006, 1, 98. 17406218 10.1038/nprot.2006.15

[70] Y. Guo , D. Li , S. Zhang , Y. Yang , J.‐J. Liu , X. Wang , C. Liu , D. E. Milkie , R. P. Moore , U. S. Tulu , D. P. Kiehart , J. Hu , J. Lippincott‐Schwartz , E. Betzig , D. Li , Cell 2018, 175, 1430. 30454650 10.1016/j.cell.2018.09.057

[71] H. Yang , Y. Wang , L. Li , F. Li , Y. He , J. Wu , C. Wei , J. Agric. Food Chem. 2019, 67, 5465. 30916943 10.1021/acs.jafc.9b00195

[72] O. Ducoudret , A. Diakov , S. Muller‐Berger , M. F. Romero , E. Fromter , Pflugers Archiv 2001, 442, 709. 11512027 10.1007/s004240100594

[73] B. Montes‐Luz , A. C. Conrado , J. K. Ellingsen , R. A. Monteiro , E. M. de Souza , G. Stacey , Current Protocols 2023, 3, 766. 10.1002/cpz1.766 37196102

[74] J. Zhu , A. Bailly , M. Zwiewka , V. Sovero , M. Di Donato , P. Ge , J. Oehri , B. Aryal , P. Hao , M. Linnert , N. I. Burgardt , C. Lücke , M. Weiwad , M. Michel , O. H. Weiergräber , S. Pollmann , E. Azzarello , S. Mancuso , N. Ferro , Y. Fukao , C. Hoffmann , R. Wedlich‐Söldner , J. Friml , C. Thomas , M. Geisler , Plant Cell 2016, 28, 930. 27053424 10.1105/tpc.15.00726 PMC4863381

[75] K. Sun , F. Lu , P.‐W. Huang , M.‐J. Tang , F.‐J. Xu , W. Zhang , J.‐Y. Zhou , P. Zhao , Y. Jia , C.‐C. Dai , Plant Cell Environ. 2022, 45, 1813. 35274310 10.1111/pce.14304

[76] Z. Du , X. Zhou , Y. Ling , Z. Zhang , Z. Su , Nucleic Acids Res. 2010, 64, 38. 10.1093/nar/gkq310 PMC2896167 20435677

[77] T. Tian , Y. Liu , H. Yan , Q. You , X. Yi , Z. Du , W. Xu , Z. Su , Nucleic Acids Res. 2017, 45, 122. 10.1093/nar/gkx382 PMC5793732 28472432

[78] M. A. Larkin , G. Blackshields , N. P. Brown , R. Chenna , P. A. McGettigan , H. McWilliam , F. Valentin , I. M. Wallace , A. Wilm , R. Lopez , J. D. Thompson , T. J. Gibson , D. G. Higgins , Bioinformatics 2007, 23, 2947. 17846036 10.1093/bioinformatics/btm404

[79] S. Kumar , G. Stecher , M. Li , C. Knyaz , K. Tamura , Mol. Biol. Evol. 2018, 35, 1547. 29722887 10.1093/molbev/msy096 PMC5967553

[80] R. P. Hellens , A. C. Allan , E. N. Friel , K. Bolitho , K. Grafton , M. D. Templeton , S. Karunairetnam , A. P. Gleave , W. A. Laing , Plant Methods 2005, 1, 13. 16359558 10.1186/1746-4811-1-13 PMC1334188

[81] T. Wang , J. Guo , Y. Peng , X. Lyu , B. Liu , S. Sun , X. Wang , Science 2012, 374, 65. 10.1126/science.abh2890 34591638

[82] A. Saleh , R. Alvarez‐Venegas , Z. Avramova , Nat. Protoc. 2008, 3, 1018. 18536649 10.1038/nprot.2008.66

[83] J. Qin , F. Wang , Q. Zhao , A. Shi , T. Zhao , Q. Song , W. Ravelombola , H. An , L. Yan , C. Yang , M. Zhang , Front. Plant Sci. 2022, 13, 882732. 35783963 10.3389/fpls.2022.882732 PMC9244705

