# Supplementary material for: Soybean Auxin Transporter PIN3 Regulates Nitrate Acquisition to Improve Nitrogen Use and Seed Traits
Source: Adv Sci (Weinh). 2025 Nov 18;13(12):e11907. doi: 10.1002/advs.202511907 (PMC12948193; doi:10.1002/advs.202511907)
Supplement: Supplementary file 1 — Supporting Information [file ADVS-13-e11907-s004.docx]

**Supporting Information**

**Soybean auxin transporter PIN3 regulates nitrate acquisition to improve nitrogen use and seed traits**

*Huifang Xu, Shiyu Huang, Jie Wang, Tian Wang, Qingqing Han, Kexin Wu, Zhen Gao, Xiaolei Shi, Tianli Tu, Ming Wang, Laimei Huang, Jiaomei Chen,* *Yunqi Liu,* *Yumei Zhang, Guoqiang Lin,* *Zhichang Chen and* *Xu Chen**


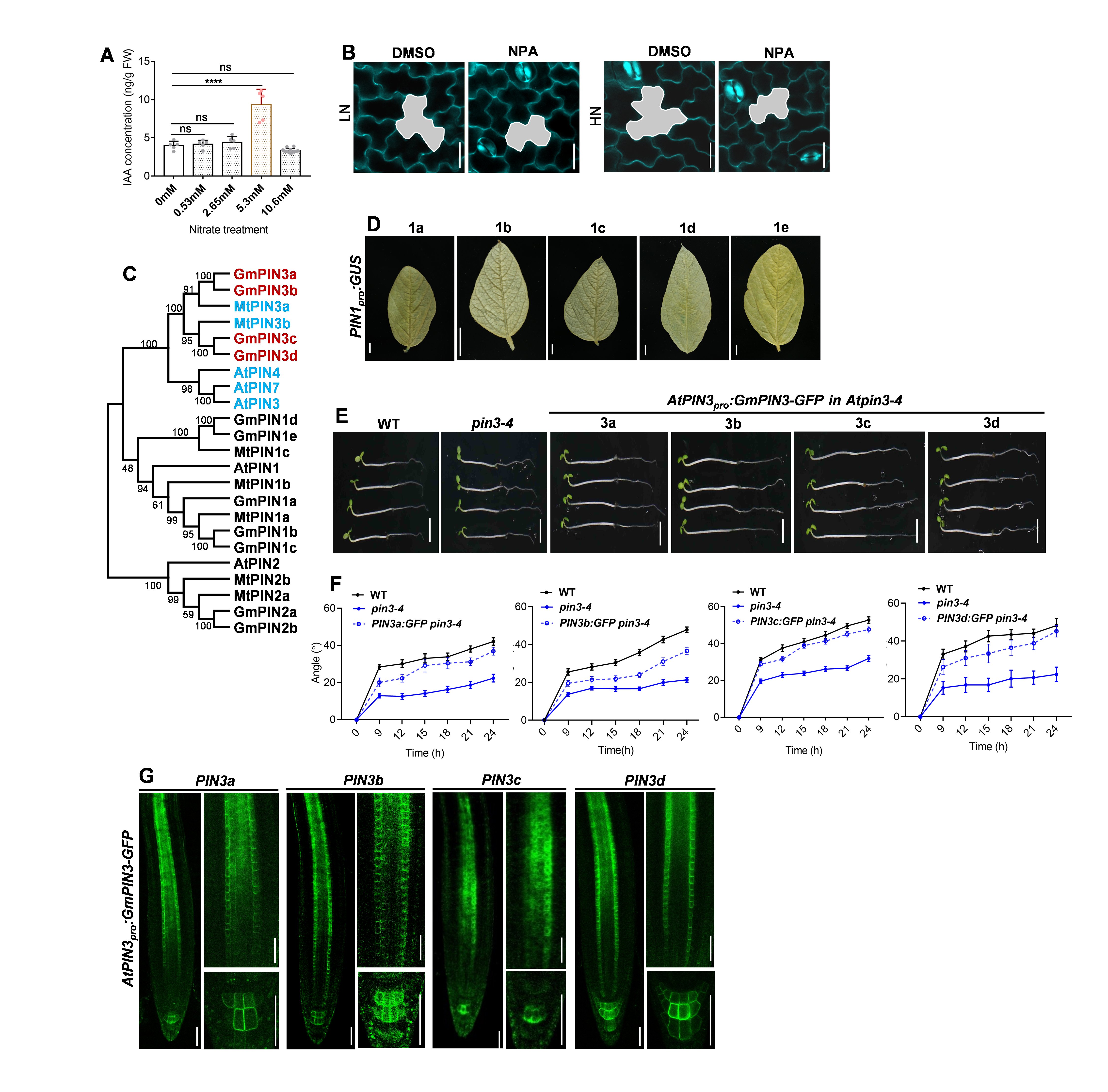


Figure S1. Soybean PIN3a and PIN3b transport auxin for leaf growth.

(A) Quantification of IAA in WT leaves by UPLC after dose-concentration nitrate treatment. Data are the mean ± SD of n = 5-14 for each genotype. P-values were determined by one-way ANOVA with Dunnett’s multiple comparisions test (ns, non-significant; ****p < 0.0001).

(B) Leaf pavement cells from 6-day-old WT plants were grown in high- or low-nitrogen conditions for 3 days, then sampled 2 days after treatment with 10 μΜ NPA before observation by light microscopy. Representative cells were individually tracked. Scale bar = 20 μm.

(C) Phylogenetic tree of PIN proteins from *Arabidopsis thaliana* (At), *Glycine max* (Gm) and *Medicago truncatula* (Mt). Numbers indicate the bootstrap replicates of gene sequences.

(D) Histochemical analysis of GUS expression in leaves from *PIN1a–e_pro_:GUS* reporter lines in the WT background grown in soil under low-nitrate conditions. Scale bar = 0.5 cm.

(E, F) Restoration of the Arabidopsis *pin3-4* hypocotyl-gravitropism mutant phenotype by *GmPIN3a–d*. *AtPIN3_pro_:GmPIN3–GFP* constructs were introduced into the *pin3-4* (Col-0) background and 5-day-old seedling phenotypes are shown in (E). Gravitropic growth angles of hypocotyl apical hooks were measured in (F). Scale bar in E = 5 mm.

(G) Confocal microscopy of GmPIN3–GFP distribution and polarity in roots of transgenic Arabidopsis *AtPIN3_pro_:GmPIN3–GFP* lines as in G. Pictures with magnification were presented in each right panel. Scale bar = 50 μm.


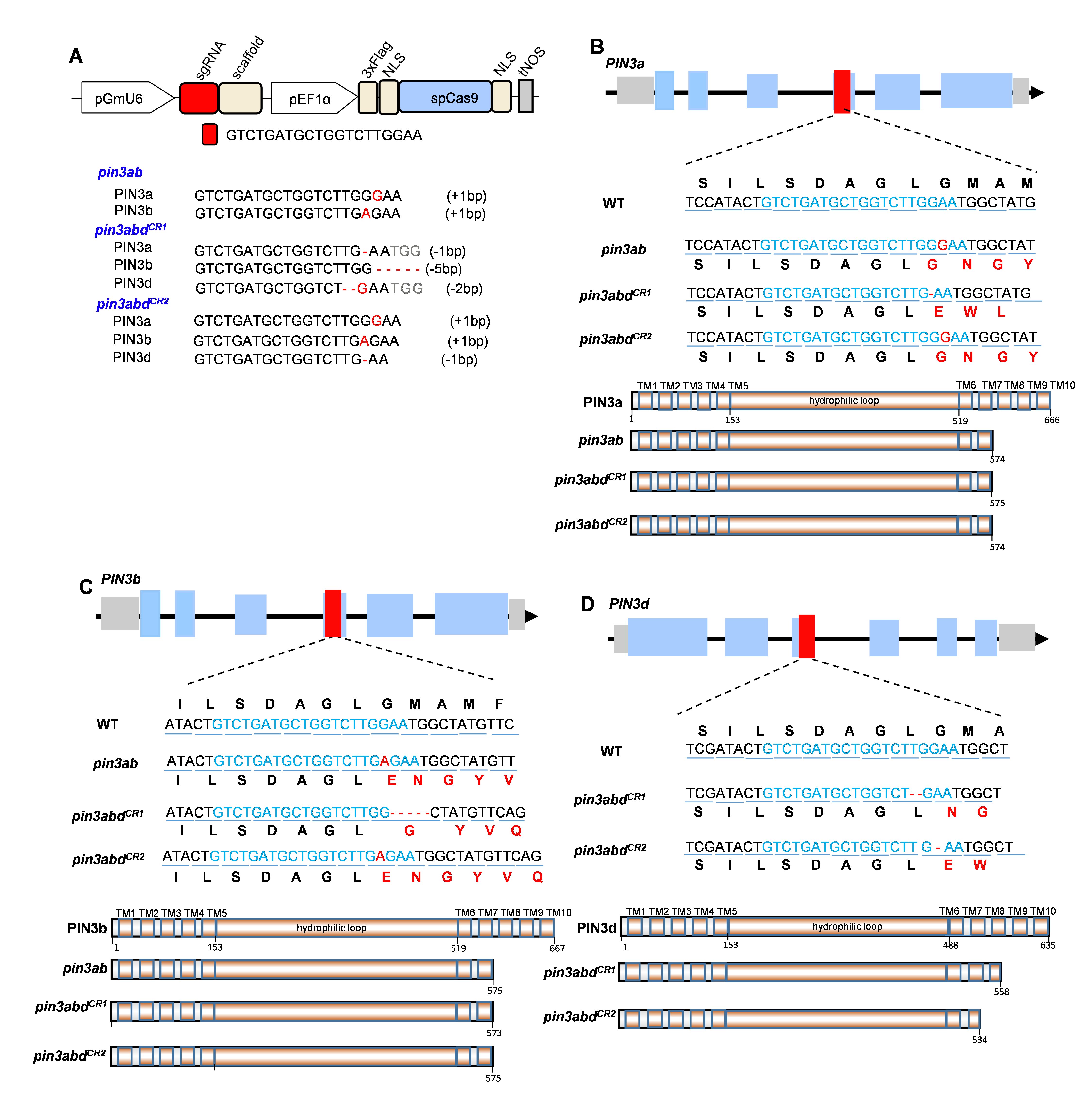


Figure S2. Characterization of *pin3ab* and *pin3abd^CR^* mutants generated by CRISPR–Cas9 gene editing.

(A) sgRNAs for CRISPR–Cas9 editing of *PIN3a*, *PIN3b* and *PIN3d*. Top: Schematic of the T-DNA region of the construct used for gene editing. Bottom: Target sites of the sgRNA in the exons of each gene were labeled in red. Dashed lines indicate the nucleotides with deletion.

(B-D) *PIN3a*, *PIN3b* and *PIN3d* genomic regions with exons depicted as blue boxes and introns as black lines. Alignments of genomic DNA carrying the CRISPR–Cas9-directed mutations in WT, *pin3ab* double mutant and two *pin3abd* triple mutants (designated CR1 and CR2) are shown in the sequences beneath. DNA sequences of the sgRNA-targeted sequence are in blue and mutated sequences are in red. Amino-acid alignments of WT versus *pin3ab* or *pin3abd^CR^* mutants are shown beneath and the changed amino acids are highlighted in red. Mutations in PIN3 genes were shown in WT, *pin3ab* and *pin3abd^CR^* mutants. In lower panels, full-length PIN3 proteins with annotated domains were presented for WT and the mutant forms of *PIN3a*, *PIN3b* and *PIN3d* in each mutant are indicated individually.


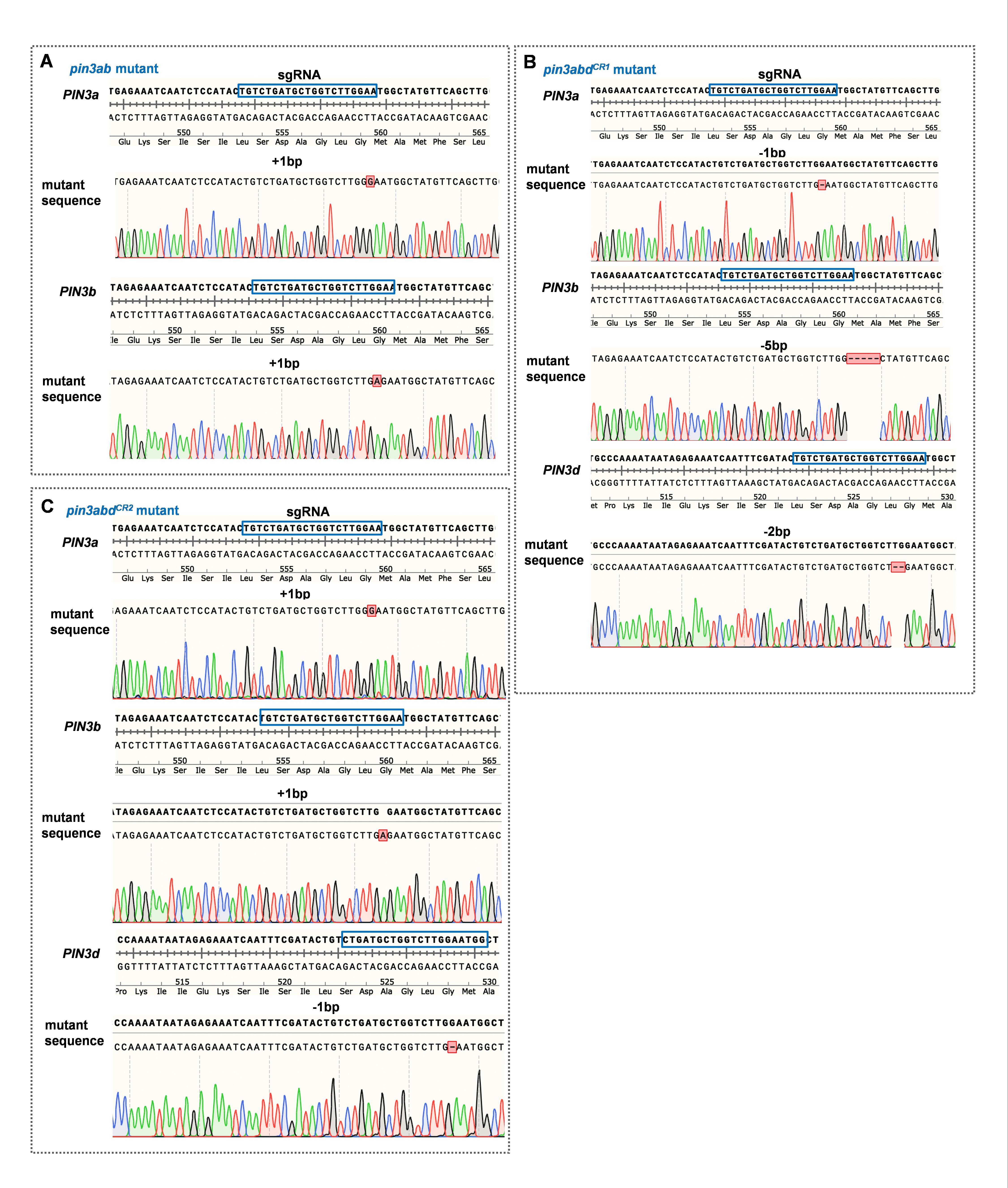


Figure S3. Strategy to generate *pin3ab* and *pin3abd^CR^* mutants by CRISPR–Cas9 gene editing.

(A-C) Sanger sequencing chromatograms with mutations in *pin3ab* and *pin3abd^CR^* mutants.


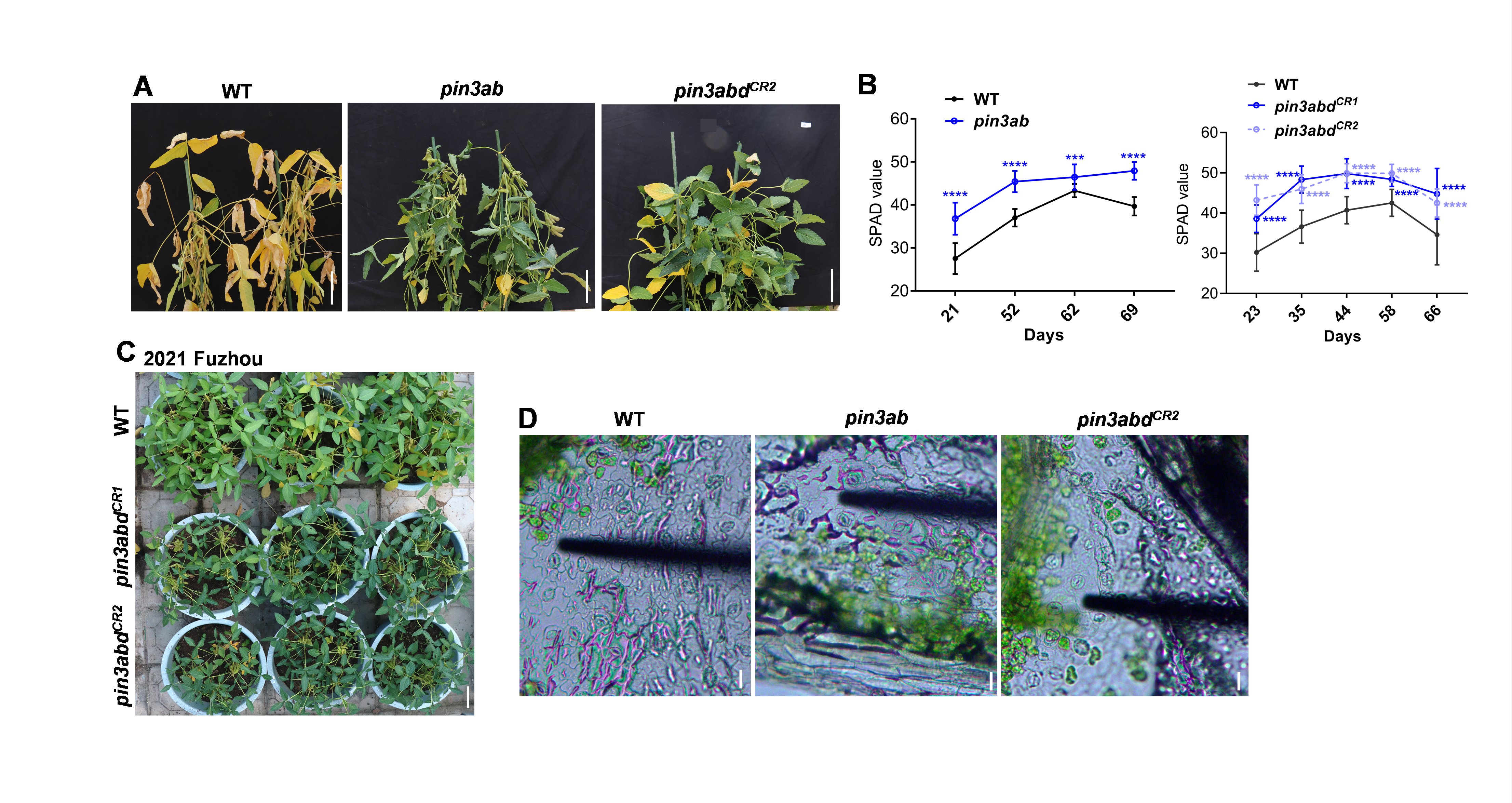


Figure S4. Phenotypic analysis of *pin3ab* and *pin3abd^CR^* mutants.

(A, B) Shoot architecture of 55-day-old WT, *pin3ab* and *pin3abd^CR^* mutant plants grown outdoors (A). SPAD values are measured at the indicated time points (B). Data are the mean ± SD of n ≧ 22 samples. Scale bars = 10 cm (A).

(C) Shoot architecture of 63-day-old WT, *pin3ab* and *pin3abd^CR^* mutant plants grown outdoors in Fuzhou (2021). Scale bars = 10 cm.

(D) Representative images of NMT measurements of IAA efflux capacity in 7-day-old WT, *pin3ab* and *pin3abd^CR^* epidermal leaf cells. The black cylinders in each image are NMT needles. Scale bar = 25 μm. P-values were determined by two-way ANOVA with Sidak multiple-comparisons (left panel) and Tukey’s multiple-comparisons (right panel) (B) (*** p < 0.01; **** p < 0.0001).


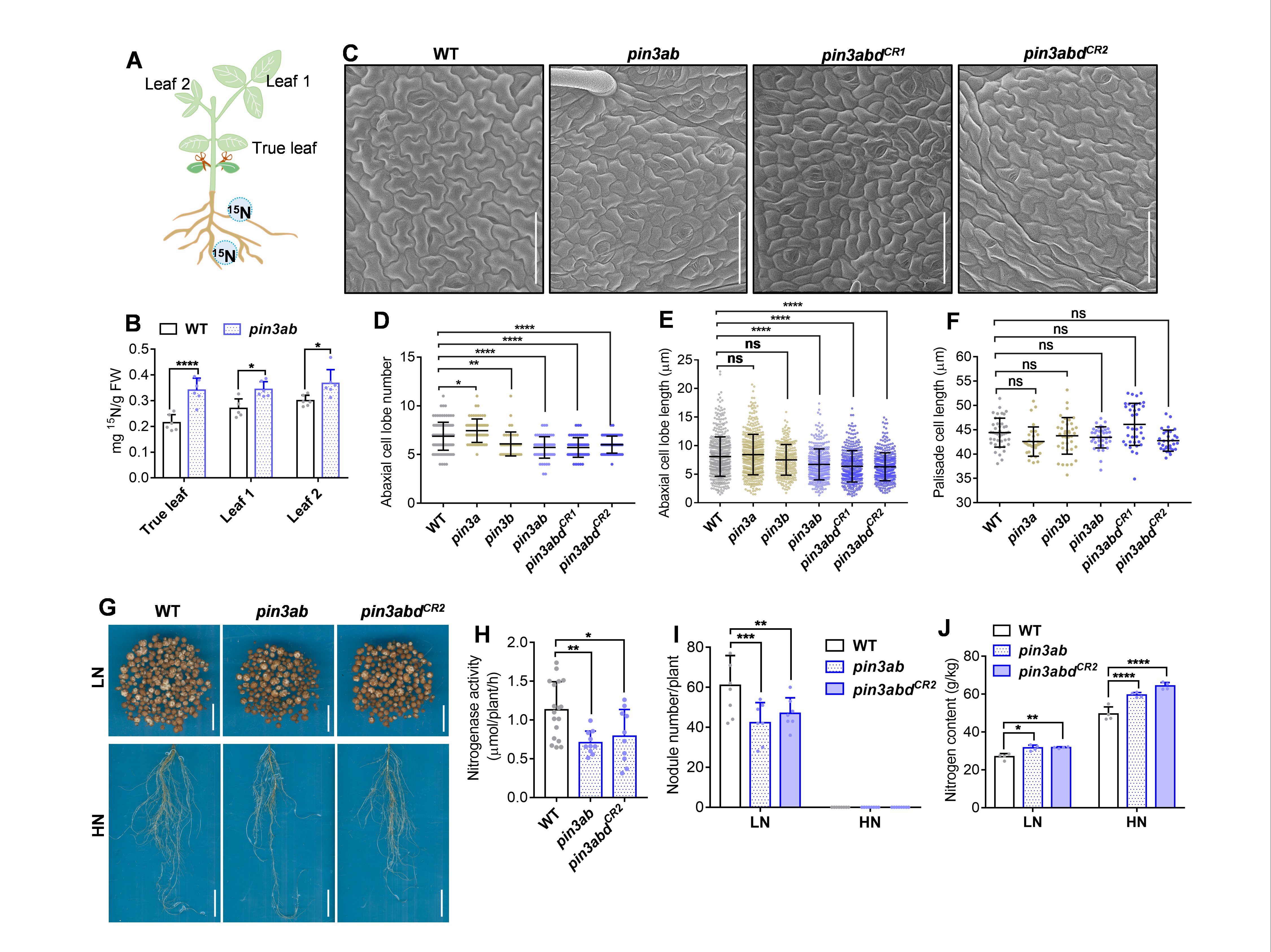


Figure S5. Pavement-cell phenotypes of *pin3ab*, *pin3abd* mutants and transgenic *pin3ab*-complemented lines.

(A, B) Summary of treatment scheme and ^15^N quantification at leaf level. 6-day-old WT and *pin3ab* plants were incubated in 5.3 mM ^15^NO_3_^-^-containing solution for 24 h; Afterward, seedlings were transferred to low-nitrogen-containing buffer for another 10 days, and ^15^N was measured in the indicated leaf positions (A). Data are the mean ± SD of n ≧ 5 samples. P values were determined by two-way ANOVA with Tukey’s multiple-comparisons test (* p < 0.05; **** p < 0.0001) (B).

(C) Leaf pavement cell morphology in 10-day-old WT, *pin3ab* and *pin3abd^CR^* leaves determined by SEM. Scale bar = 100 μm.

(D–F) Leaf pavement-cell lobe number (D), lobe length (E) and palisade-cell length (F) were calculated in WT, *pin3ab* and *pin3abd^CR^* plants. Data are the mean ± SD of n = 116, 62, 45, 64, 65 and 62 samples (D), n = 505, 463, 270, 367, 373 and 373 samples (E) and n = 42, 32, 38, 37, 37, 31 (F) from left to right.

(G–J) In WT, *pin3ab* and *pin3abd^CR2^* mutants after rhizobia inoculation, nodule phenotype under low nitrate and root morphology under high nitrate were shown (G), nitrogenase activity was detected under low nitrate condition (H), nodule number (I) and nitrogen content (J) were measured under both low and high nitrate conditions. Data are the mean ± SD of n = 10–20 (H), 8–10 (I) and 4 (J) replicates per genotype.

P-values were determined by one-way ANOVA with Dunnett’s multiple-comparisons test (D, E, F, H) and two-way ANOVA with Sidak’s or Tukey’s multiple-comparisons test (I, J) (* p < 0.05; ** p < 0.01; *** p < 0.001; **** p < 0.0001; ns, non-significant). Scale bar = 1 cm (upper panels) and 5cm (lower panels) in G.


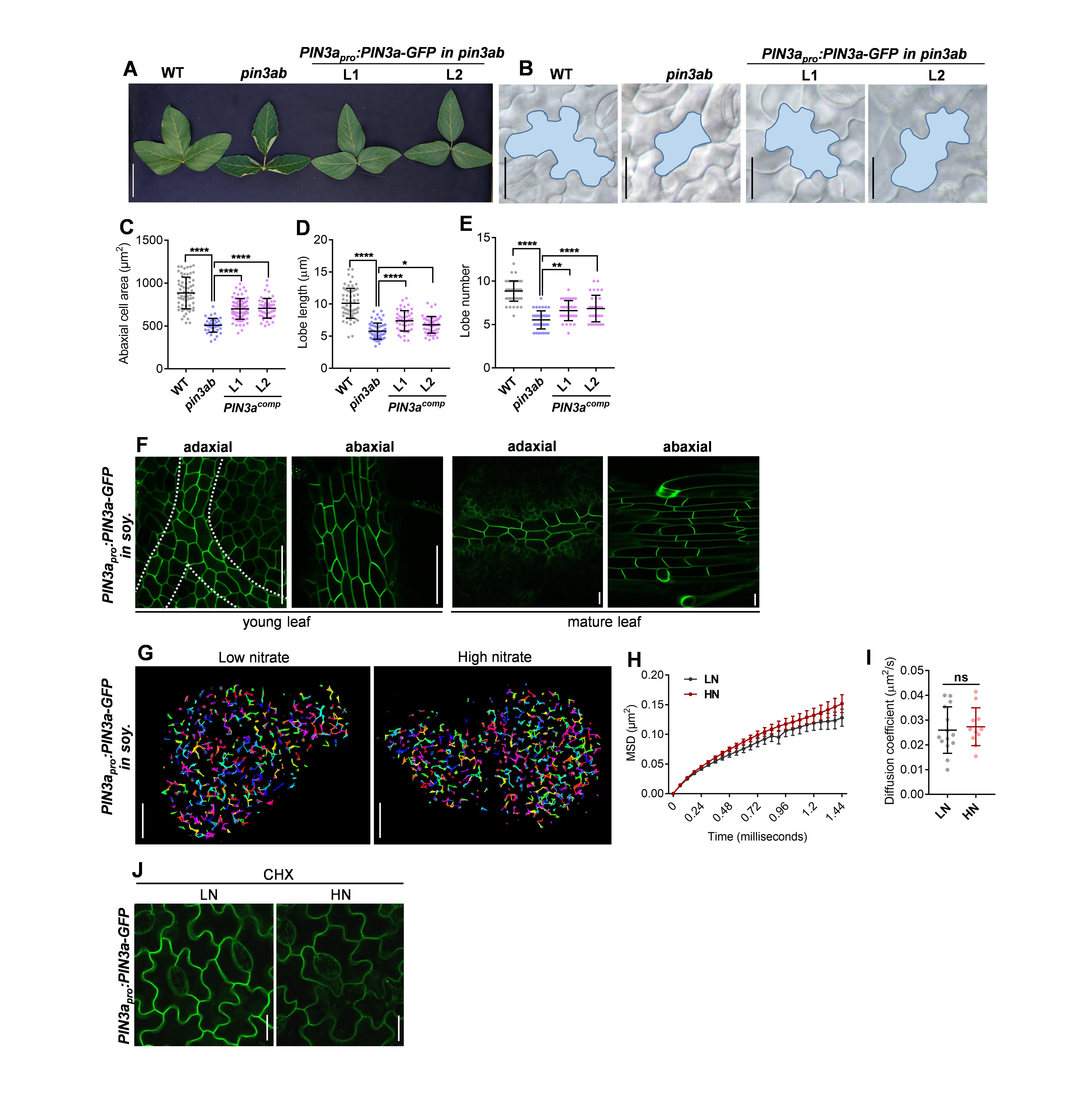


Figure S6. GmPIN3a–GFP localizes to pavement-cell junctions.

(A–E) Leaf phenotypes (A) and pavement-cell shapes (B) of 25-day-old WT, *pin3ab* and two independent transgenic *PIN3a_pro_:PIN3a–GFP* (*pin3ab* background) complementing lines (*‘PIN3acomp’*). Quantification of pavement-cell area (C), lobe length (D) and lobe number (E). Data are the mean ± SD of n = 66, 42, 75, and 51 samples (C), n = 63, 66, 47 and 51 samples (D) and n = 50, 39, 33 and 32 samples (E) from left to right. Scale bar in A = 2 cm and B = 20 μm. P-values were determined by one-way ANOVA with Tukey’s multiple-comparisons test (C–E) (* p < 0.05; ** p < 0.01; **** p < 0.0001).

(F) Confocal analysis of PIN3a–GFP localization in adaxial and abaxial vasculature of 5- and 11-day-old *PIN3a_pro_:PIN3a–GFP* transgenic soybean leaves grown in vermiculite under low-nitrate conditions. Scale bar = 20 μm.

(G–I) Analysis of PIN3a–GFP foci on the plasma membrane recorded by Multimodality Structured Illumination Microscopy (Mutli-SIM). Six-day-old *PIN3a_pro_:PIN3a–GFP* soybean plants were transferred to low- and high-nitrate conditions for 5 d before imaging. Trajectories of the PIN3a–GFP particles during time-lapse imaging for 0.05 s (G). Mean-square displacement (MSD) of PIN3a–GFP particles were plotted as a function of time at 0.1 s resolution (H). Data are the mean ± SD of n = 14 and 11 regions. For each ROI, the averaged MSD was used to quantify mean diffusion coefficients (I). Student’s t-test test was used to compare means in I (ns, non-significant). Scale bar in (G) = 5 μm.

(J) 6-day-old *PIN3a_pro_:PIN3a–GFP* stable-transgenic plants were transferred to low- or high-nitrogen conditions for additional 5 days, then were co-treated with 100 μm cycloheximide (CHX) for 6 h.


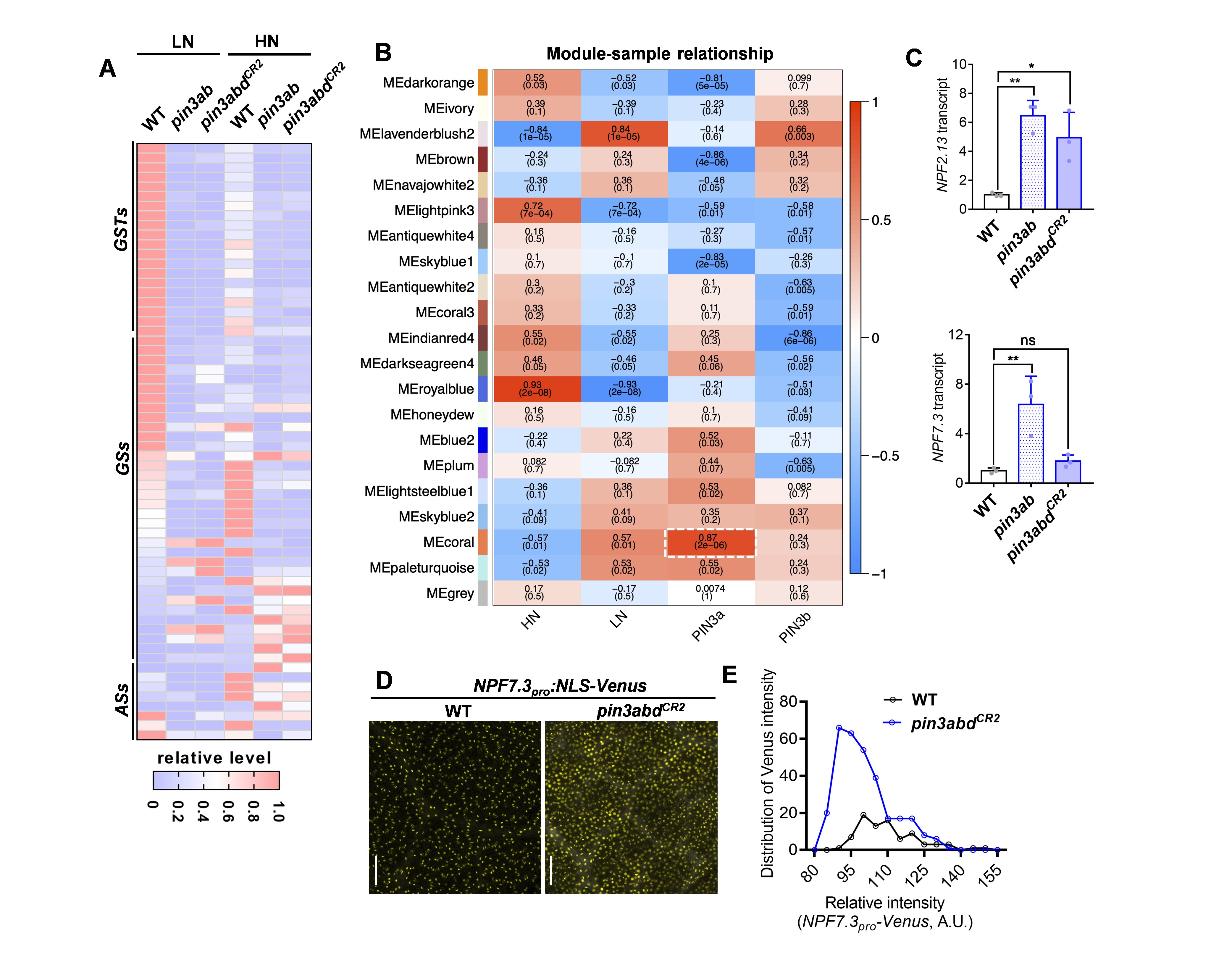


Figure S7. Transcriptome analysis of soybean *pin3* mutants.

(A) Heatmap depicting relative expression of *GLUTAMINE SYNTHETASEs (GSs)*, *GLUTATHIONE S-TRANSFERASEs (GSTs)* and *ASPARAGINE SYNTHETASEs (ASs)* after transfer to high- or low-nitrate conditions from transcriptome data.

(B) WGCNA transcriptome analysis for WT, *pin3ab* and *pin3abd^CR^* leaves upon low- or high-nitrate treatment based on expression categories. Module analysis of high-nitrate-, low-nitrate-, *PIN3a-* or *PIN3b-*coexpression network. Low to high relationship of each module is indicated according to bar color. White dotted frames highlight the highest co-expression module for PIN3a.

(C) RT–qPCR of *NPF2.13* and *NPF7.3* expression in 11-day-old WT, *pin3ab* and *pin3abd^CR2^* leaves. Data are the mean ± SD of n = 3. ACT11 was used as a reference gene for normalization. P-values were determined by one-way ANOVA with Dunnett’s multiple comparisions test (* p < 0.05; ** p < 0.01; ns, non-significant).

(D, E) Confocal analysis of Venus signal in transgenic soybean leaves expressing *NPF7.3_pro_:NLS–Venus* in WT and *pin3abd^CR2^* backgrounds (D). Venus signal was measured in each nuclear discrete value (E, n = 82 and 308 from 15 independent pictures; AU, arbitrary unit). Scale bar in (D) = 10 μm.


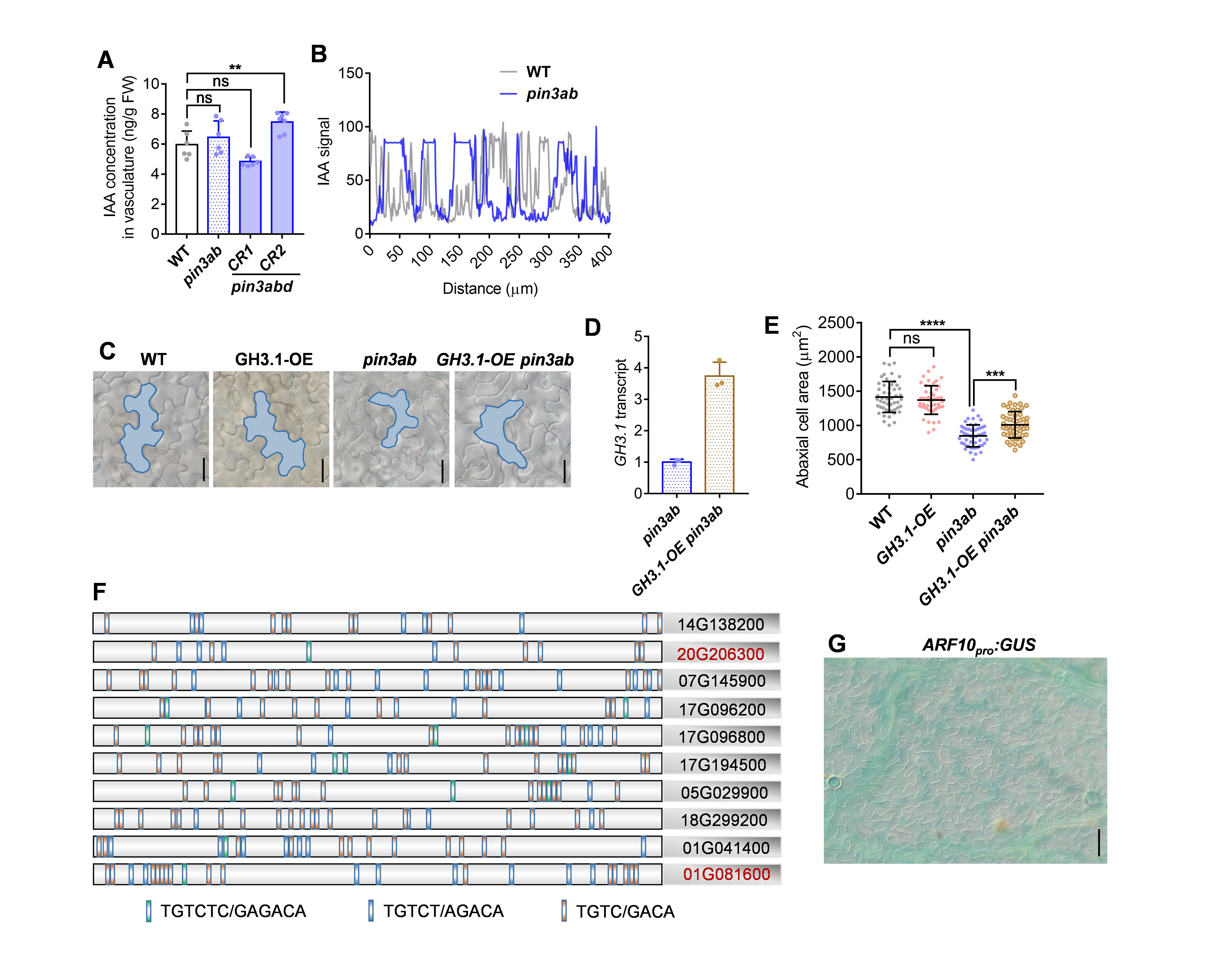


Figure S8. *NRT* expression and auxin contents are increased in *pin3ab* and *pin3abd* mutants.

(A) Quantification of IAA in vasculature by UPLC in 10-day-old WT, *pin3ab* and *pin3abd^CR^* mutants. Data are the mean ± SD of n = 6 or 7 for each genotype. P-values were determined by one-way ANOVA with Fisher’s LSD test (** p < 0.01; ns, non-significant).

(B) Anti-IAA antibody immunostaining of auxin distribution in WT and pin3ab leaf pavement cells. IAA signal was tracked as profile according to the orange lines in Figure 5B.

(C-E) Leaf pavement cells from 22-day-old WT, over-expressing *GH3.1*, *pin3ab*, over-expressing *GH3.1* in the *pin3ab* mutant plants were observed by light microscopy. Representative cells were individually tracked (C) and quantification of pavement-cell area (E). Data are the mean ± SD of n = 50 samples (E). Scale bar in C = 20 μm. P-values were determined by one-way ANOVA with Tukey’s multiple-comparisons test (*** p < 0.001; **** p<0.0001; ns, non-significant). Characterization of transgenic lines over-expressing *GH3.1* in the *pin3ab* mutant background. *GH3.1* expression level was detected by RT–qPCR. Data are the mean ± SD of n = 3.

(F) Summary of ARF-binding TGTC(TC) cis-elements found in promoters (3 kb) of soybean NPF genes. *Glyma.01g081600 (NPF2.13)* and *Glyma.20g206300 (NPF7.3)* are highlighted in red.

(G) Visualization of GUS signal in transgenic soybean leaves expressing *ARF10_pro_: GUS* in WT plant. Scale bar = 50 μm.


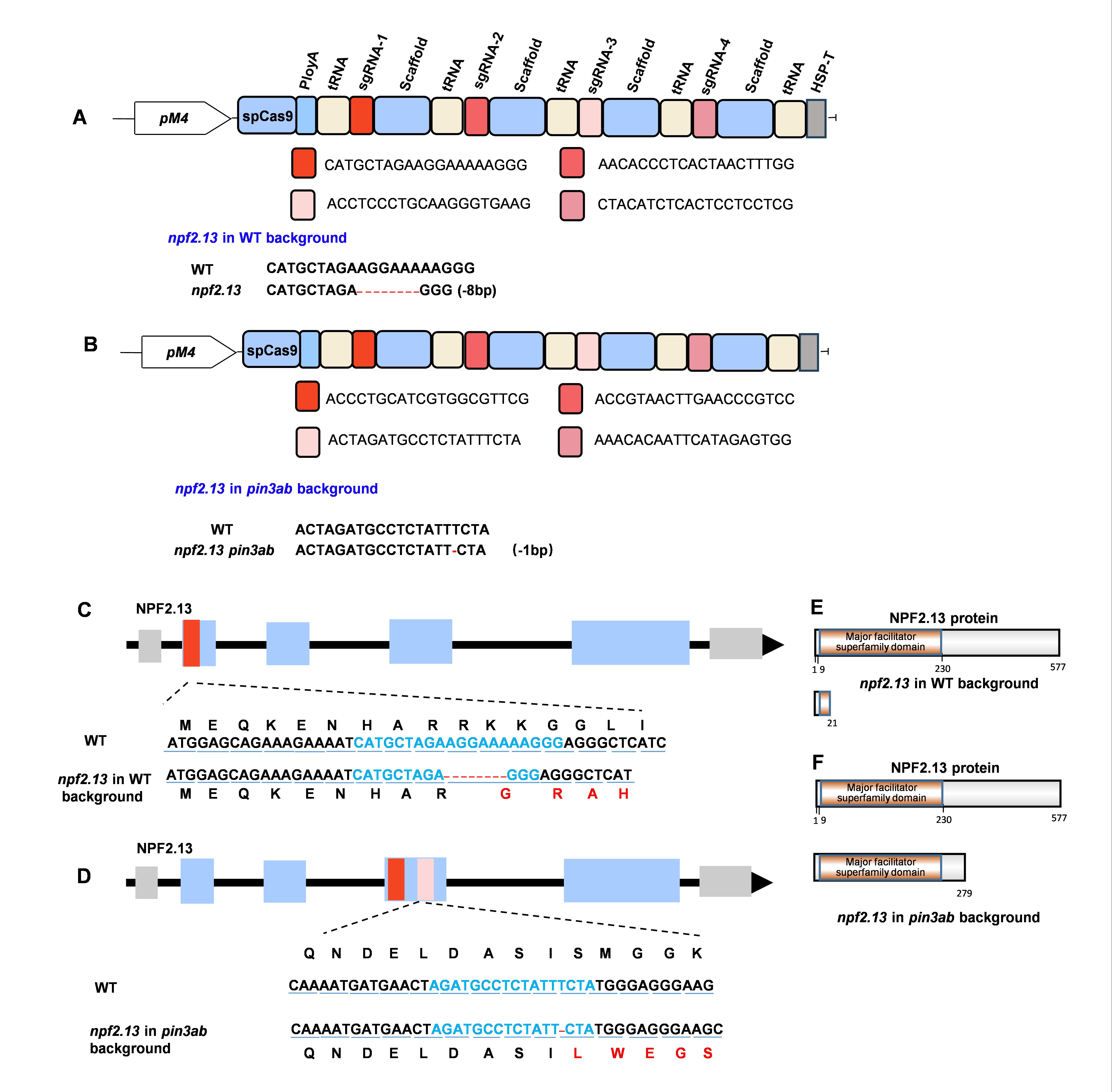


Figure S9. Strategy to generate *npf2.13* mutant in WT and *pin3ab* mutant background by CRISPR–Cas9 gene editing.

(A, B) sgRNAs for CRISPR–Cas9 editing of *NPF2.13* in WT background (A) or *pin3ab* background (B). Top: Schematic of the T-DNA region of the construct used for gene editing. Bottom: Target sites of the sgRNA in the exons of each gene were labeled in red. Dashed lines indicate the nucleotides with deletion.

(C, D) *NPF2.13* genomic regions with exons depicted as blue boxes and introns as black lines. Alignments of genomic DNA carrying the CRISPR–Cas9-directed mutations in WT background (C) or *pin3ab* background (B). Mutants’ information is shown in the sequences beneath. DNA sequences of the sgRNA-targeted sequence are in blue and mutated sequences are in red. Amino-acid alignments of WT versus *npf2.13* mutant is shown beneath and the changed amino acids are highlighted in red.

(E) Full-length NPF2.13 proteins with annotated domains were presented for WT and the mutant forms of *npf2.13* (WT background) mutant is indicated.

(F) Full-length NPF2.13 proteins with annotated domains were presented for WT and the mutant forms of *npf2.13* (*pin3ab* background) mutant is indicated.


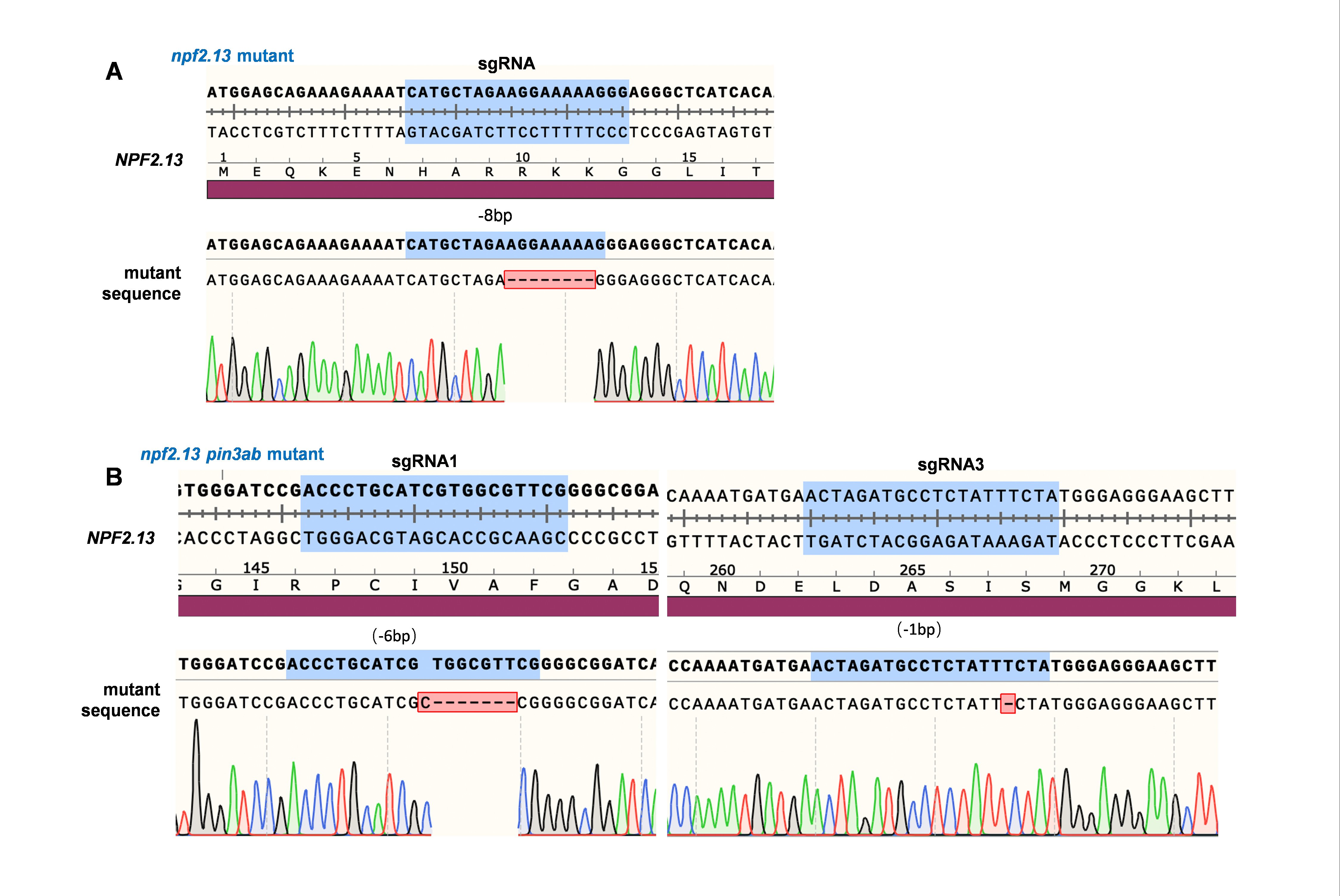


Figure S10. Strategy to generate a *npf2.13* mutant in WT and *pin3ab* mutant background by CRISPR–Cas9 gene editing.

(A) Sanger sequencing chromatograms of mutations detected in the *npf2.13* (WT background) mutant.

(B) Sanger sequencing chromatograms of mutations detected in the *npf2.13* (*pin3ab* background) mutant. In the *pin3ab* background, the sgRNA1 editing event induces a 6-base deletion in the *NPF2.13* gene, resulting in two code deletion; The sgRNA3 editing event introduces a 1-base deletion in *NPF2.13*.


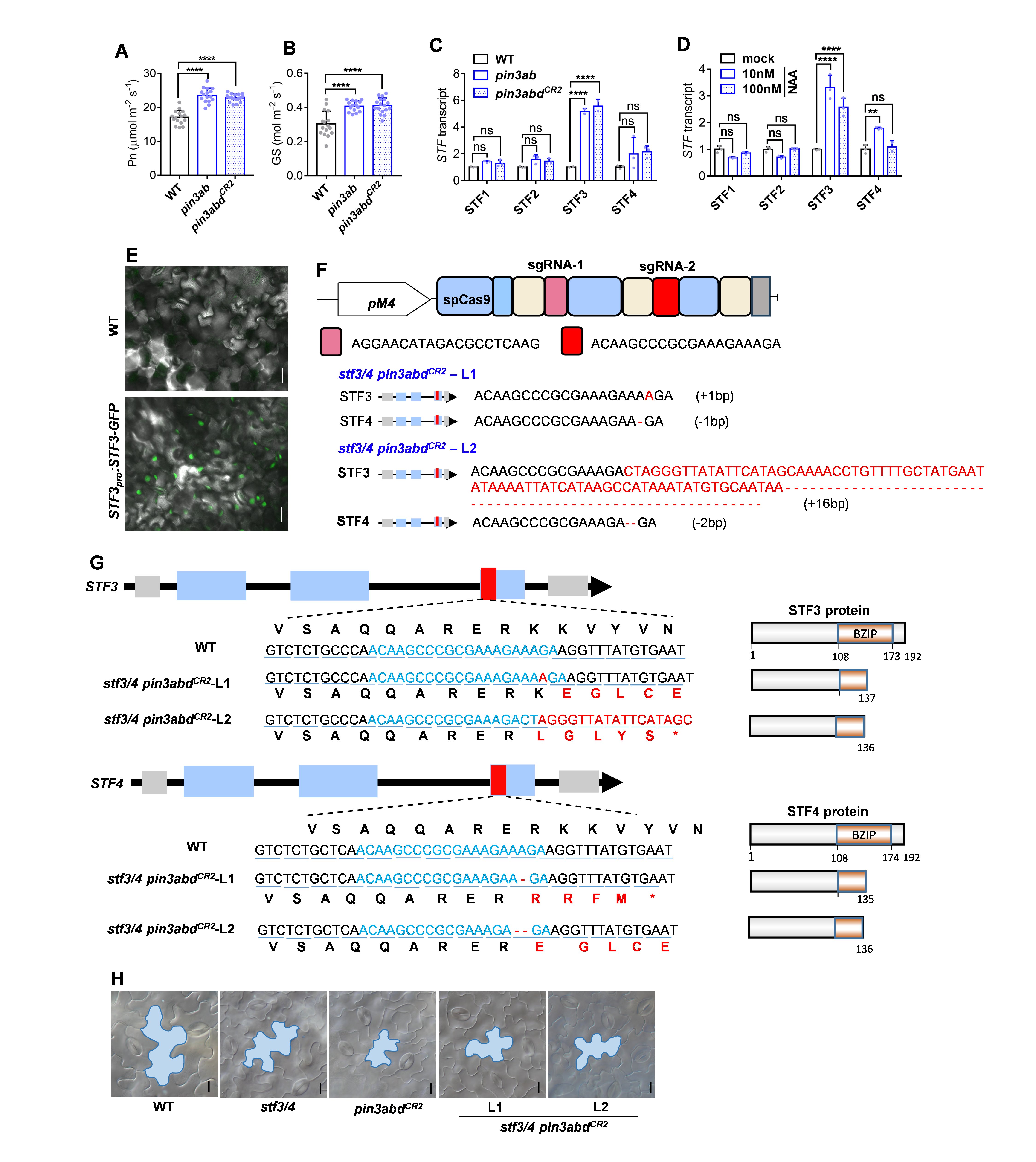


Figure S11. Generation of *stf3/4* mutants in the *pin3abd^CR2^* background by CRISPR–Cas9 gene editing.

(A, B) Photosynthesis parameters in 45-day-old WT, *pin3ab* and *pin3abd^CR2^* mutants. Net photosynthetic rate (Pn, A) and stomatal conductance (Gs, B). Data are the mean ± SD of n = 16–17 plants. P-values were determined by one-way ANOVA with Dunnett’s multiple-comparisons test (****p < 0.0001).

(C, D) RT–qPCR of *STF1–4* expression in 11-day-old WT, *pin3ab* and *pin3abd^CR2^* leaves (C), and RT–qPCR of *STF1–4* expression in WT leaves upon NAA treatment (D). Data are the mean ± SD of n = 3. *ACT11* was used as a reference gene for normalization. P-values were determined by two-way ANOVA with Tukey’s multiple-comparisons (** p < 0.01; **** p < 0.0001; ns, non-significant).

(E) Confocal-microscopy images of STF3–GFP subcellular localization in leaves on the abaxial surface from *STF3_pro_:STF3*–*GFP* stable-transgenic soybean plants. WT plants without fluorescence was used as a negative control. Scale bars = 20 μm.

(F) Top: Summary of the T-DNA region used for editing *STF3* and *STF4* via two sgRNAs. Middle: DNA sequence of the sgRNA-target sequences in black and mutation sequences in red. Bottom: Alignments of genomic DNA carrying the CRISPR–Cas9-directed mutations in WT and *stf3/4* mutants (line 1 and line 2) are shown in the sequence beneath.

(G) Mutations in *STF3/4* genes in *stf3/4* *pin3abd^CR2^*-L1 and -L2 mutants. Full-length STF3 and STF4 proteins are presented in WT and the mutated forms expressed in each allele are indicated individually. Top: Schematics of *STF3* and *STF4* genomic regions with exons depicted as blue boxes and introns as black lines. Bottom: Amino-acid alignment of STF3 and STF4 sequences in WT and *stf3/4* mutants with the changed amino acids in red. In right panels, full-length STF3/4 proteins with annotated domains were presented for WT and the mutant forms of STF3 and STF4 in each mutant are indicated individually.

(H) Leaf pavement cells of WT, *stf3/4*, *pin3abd^CR2^* and two independent *stf3/4 pin3abd^CR2^* quintuple mutants plants were observed by light microscopy. Representative cells were individually tracked. Scale bar in H = 20 μm.


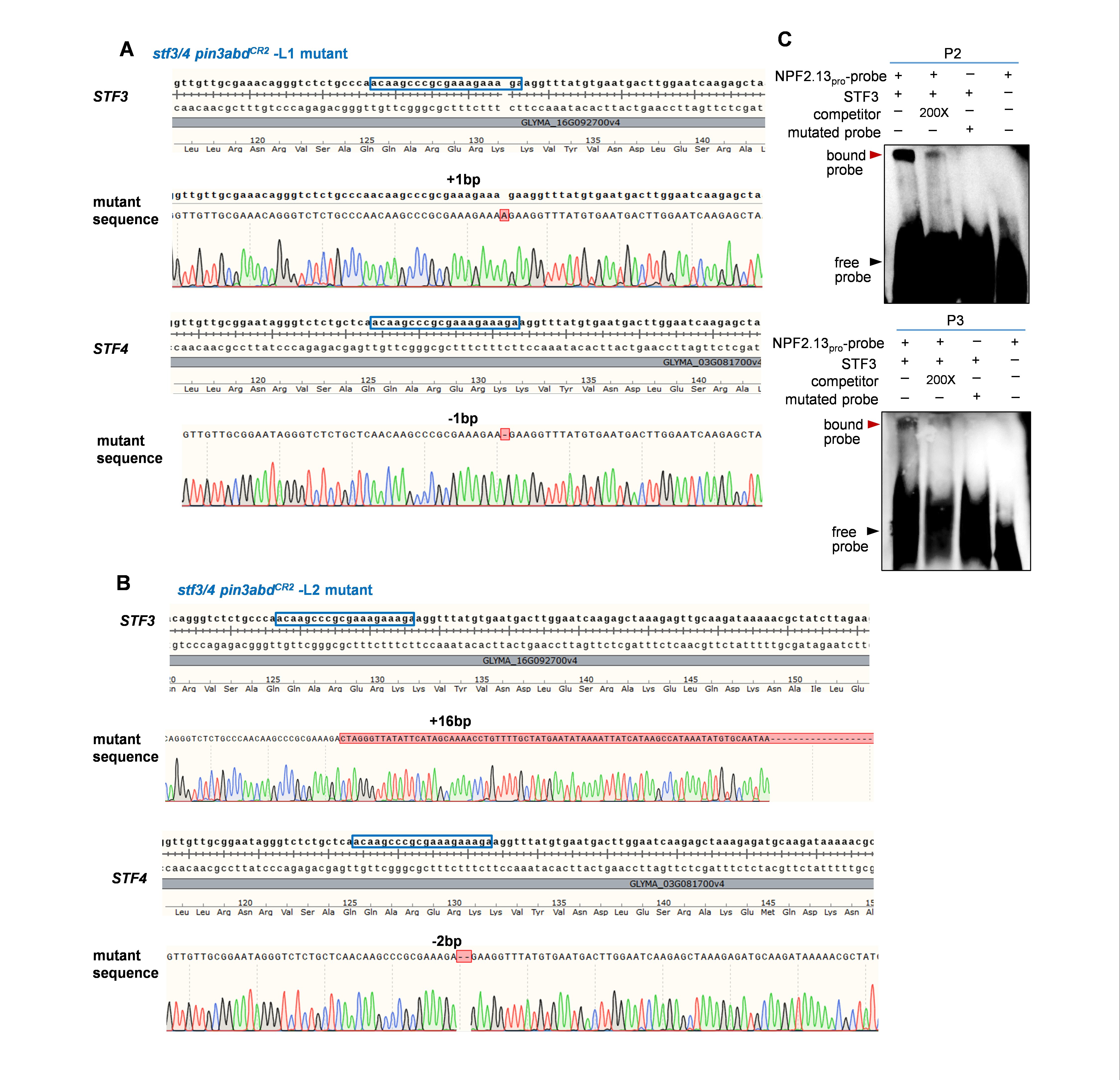


Figure S12. Genetic analysis of *stf3/4 pin3abd^CR2^* quintuple mutants and STF3 binding to G boxes in the *NPF2.13* promoter.

(A, B) Sanger sequencing chromatograms showing mutations in *stf3/4* mutants. Line 1 is shown in (A) and line 2 in (B).

(C) EMSA showing 6xHis–STF3 directly binds in vitro to G-box motifs located at P2 (top, 2,787 to 2,826 bp relative to the start codon) and P3 (bottom, 1,784 to 1,821bp relative to the start codon) sites in the *NPF2.13* promoter. For competition analysis, unlabeled DNA probes (competitors) were added in 200-fold (x) molar excess relative to the labeled probes and competition with unlabeled mutated probes in 1-fold excess were added.


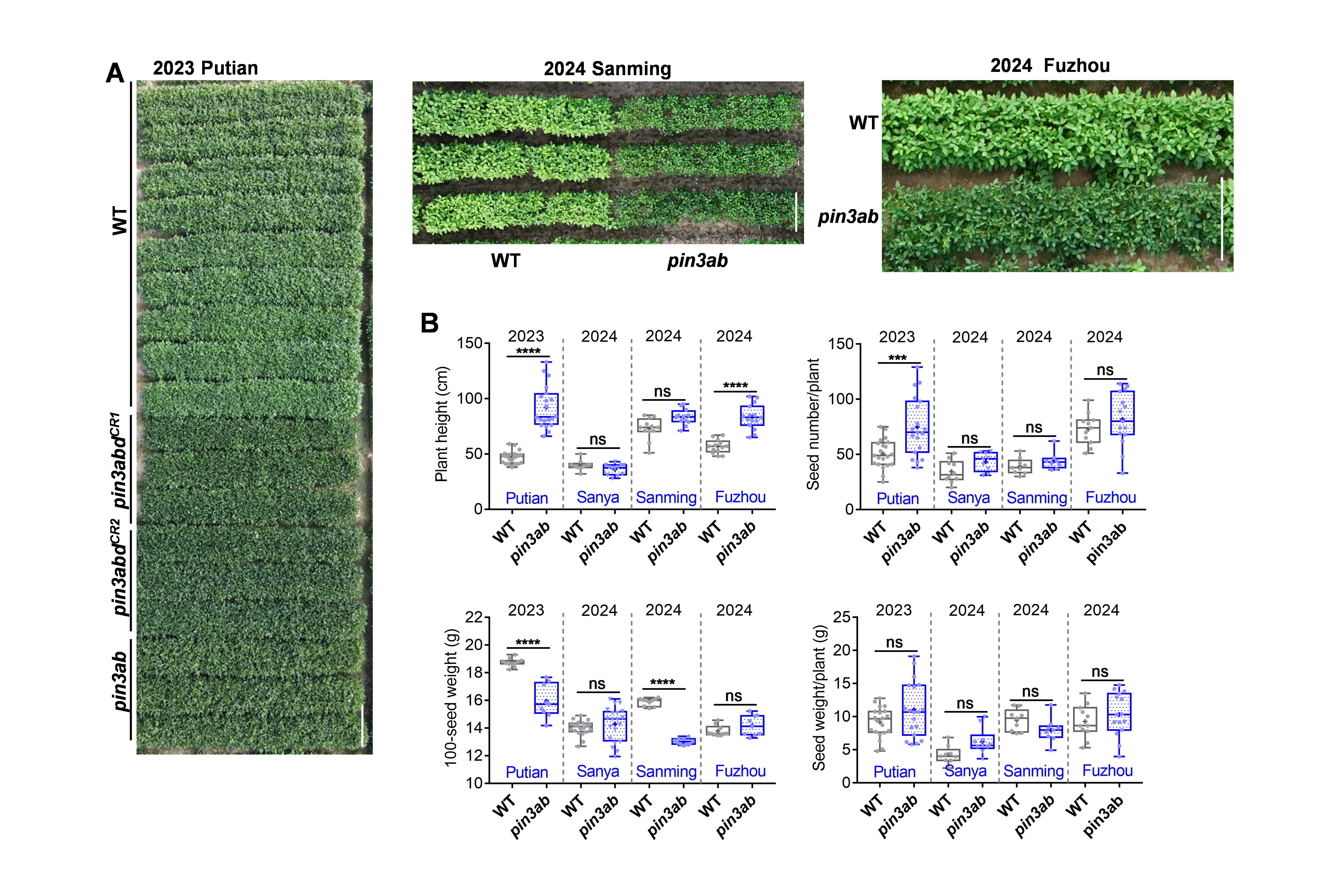


Figure S13. Phenotypic analysis of field-grown *pin3ab* mutants.

(A) Under normal nitrate-fertilizer growth conditions, an aerial view of 78-d-old WT and *pin3ab* double and *pin3abd^CR1^* and *pin3abd^CR2^* triple mutants cultivated in Putian (2023), aerial view of 50-d-old WT and *pin3ab* double mutants cultivated in Sanming (2024) and 40-d-old WT and *pin3ab* double mutants cultivated in Fuzhou (2024) was captured. Scale bars = 1 m. (B) Phenotypic analysis of field-grown WT and *pin3ab* mutants grown in Putian (2023), Sanya (2024), Sanming (2024) and Fuzhou (2024), under normal nitrate-fertilizer growth conditions. Data are the mean ± SD of n ≧ 8 plants for each genotype quantification. P-values were determined by one-way ANOVA with Turkey’s multiple-comparisons test (*** p < 0.001; **** p < 0.0001; ns, non-significant).
